# Genetic variation that determines *TAPBP* expression levels associates with the course of malaria in an HLA allotype-dependent manner

**DOI:** 10.1073/pnas.2205498119

**Published:** 2022-07-13

**Authors:** Victoria Walker-Sperling, Jean C. Digitale, Mathias Viard, Maureen P. Martin, Arman Bashirova, Yuko Yuki, Veron Ramsuran, Smita Kulkarni, Vivek Naranbhai, Hongchuan Li, Stephen K. Anderson, Lauren Yum, Robert Clifford, Hannah Kibuuka, Julie Ake, Rasmi Thomas, Sarah Rowland-Jones, John Rek, Emmanuel Arinaitwe, Moses Kamya, Isabel Rodriguez-Barraquer, Margaret E. Feeney, Mary Carrington

**Affiliations:** ^a^Laboratory of Integrative Cancer Immunology, Center for Cancer Research, National Cancer Institute, Bethesda, Maryland, 20892;; ^b^Department of Medicine, University of California San Francisco, San Francisco, California, 94158;; ^c^Department of Epidemiology and Biostatistics, University of California San Francisco, San Francisco, California, 94143;; ^d^Basic Science Program, Frederick National Laboratory for Cancer Research, National Cancer Institute, Frederick, Maryland, 21702;; ^e^School of Laboratory Medicine and Medical Sciences, College of Health Sciences, University of KwaZulu-Natal, Durban, 4041, South Africa;; ^f^Texas Biomedical Research Institute, Host Pathogen Interaction Program, San Antonio, Texas, 78227;; ^g^Dana Farber Cancer Institute, Department of Medical Oncology, Boston, Massachusetts, 02215;; ^h^MGH Cancer Center, Massachusetts General Hospital, Boston, Massachusetts, 02114;; ^i^Department of Medicine, Massachusetts General Hospital, Boston, Massachusetts, 02114;; ^j^Centre for the AIDS Programme of Research in South Africa (CAPRISA), Durban, 4041, South Africa;; ^k^Laboratory of Cancer Immunometabolism, Center for Cancer Research, National Cancer Institute, Frederick, Maryland, 21702;; ^l^U.S. Military HIV Research Program,, Walter Reed Army Institute of Research, Silver Spring, Maryland, 20910;; ^m^Henry M. Jackson Foundation for the Advancement of Military Medicine, Bethesda, Maryland, 20817;; ^n^Makerere University Walter Reed Project, Kampala, Uganda;; ^o^Viral Immunology Unit, Nuffield Department of Medicine, University of Oxford, Oxford, OX3 7FZ, UK;; ^p^Infectious Diseases Research Collaboration, Kampala, Uganda;; ^q^Department of Medicine, Makerere University, Kampala, Uganda;; ^r^Department of Pediatrics, University of California San Francisco, San Francisco, California, 94158;; ^s^Ragon Institute of MGH, MIT and Harvard, Cambridge, Massachusetts, 02139

**Keywords:** malaria, tapasin, HLA

## Abstract

HLA class I (HLA-I) allotypes vary widely in their dependence on tapasin (TAPBP), an integral component of the peptide-loading complex, to present peptides on the cell surface. We identified two single-nucleotide polymorphisms that regulate *TAPBP* messenger RNA (mRNA) expression in Africans, *rs111686073* (*G*/*C*) and *rs59097151 (A*/*G)*, located in an AP-2α transcription factor binding site and a microRNA (miR)-4486 binding site, respectively. *rs111686073G* and *rs59097151A* induced significantly higher *TAPBP* mRNA expression relative to the alternative alleles due to higher affinity for AP-2α and abrogation of miR-4486 binding, respectively. These variants associated with lower *Plasmodium falciparum* parasite prevalence and lower incidence of clinical malaria specifically among individuals carrying tapasin-dependent HLA-I allotypes, presumably by augmenting peptide loading, whereas tapasin-independent allotypes associated with relative protection, regardless of imputed *TAPBP* mRNA expression levels. Thus, an attenuated course of malaria may occur through enhanced breadth and/or magnitude of antigen presentation, an important consideration when evaluating vaccine efficacy.

## Significance

Peptides are selected and bound to HLA-I within the endoplasmic reticulum, aided by the molecular chaperone tapasin. HLA-I allotypes vary in their dependence on tapasin for peptide loading, and tapasin-independent allotypes present a more diverse set of peptides than tapasin-dependent allotypes. We show that imputed *TAPBP* messenger RNA-expression levels, along with HLA-I allotype-specific tapasin dependence level, associate with malaria outcome. High *TAPBP* expression significantly associated with protection amongst individuals with tapasin-dependent HLA allotypes relative to low tapasin expression. Tapasin expression had no effect on tapasin-independent allotypes, which conferred protection regardless of imputed tapasin-expression levels. Thus, intrinsically high tapasin-expression levels may compensate for the restrictive nature of HLA-I tapasin dependence in the peptide-loading process, attenuating the course of malaria.

Efficient antigen presentation by major histocompatibility complex (MHC) class I (MHC-I; HLA-I in humans) is integral to the CD8 T-cell-mediated adaptive immune response and natural killer cell-mediated innate immunity. The peptide-loading complex (PLC), localized within the endoplasmic reticulum (ER) and composed of transporter associated with antigen processing (TAP) proteins, ERp57, tapasin, and calreticulin, is necessary for the efficient assembly and presentation of the HLA-I:peptide complex on the cell surface (reviewed in ref. 1). Tapasin is a critical PLC component, which bridges HLA-I to TAP, acts as a chaperone stabilizing HLA-I in a peptide-receptive conformation, and promotes exchange for higher-affinity peptides (2–6). Down-modulation of tapasin function has been found in some viral infections (7, 8) and cancers (9, 10) as a mechanism for immunological escape from CD8 T-cell responses through disruption of the HLA-I peptide-loading process.

HLA-I allotypes vary markedly in their dependence on tapasin for peptide loading and expression on the cell surface (11–16) due to structural polymorphisms resulting in the differential ability of HLA-I allotypes to select peptides without tapasin assistance (2, 14, 17, 18). We have recently determined tapasin-dependence values for a large set of common HLA-I allotypes by determining the ratio of HLA-I expression levels on the cell surface in the presence vs. absence of tapasin, which showed a continuum of values for HLA-A, HLA-B, and HLA-C (11). This variation is functionally relevant, as tapasin-independent allotypes were shown to present a broader array of peptides than tapasin-dependent allotypes, and tapasin independence conferred protection in HIV-1 disease.

Given the role of tapasin in shaping the HLA-I peptide repertoire and its impact on disease outcome, we hypothesized that natural variation in its expression level could also affect disease pathogenesis and susceptibility. Two single-nucleotide polymorphisms (SNPs) were identified in Black populations (both positions are fixed in Whites) that associate significantly with *TAPBP* messenger RNA (mRNA) expression: *rs111686073*, located in an AP-2α transcription factor binding site in the 5′ untranslated region (UTR) of the *TAPBP* gene, and *rs59097151*, located in an hsa–microRNA (miR)-4486 binding site in the 3′ UTR of the *TAPBP* gene. Among subjects with tapasin-dependent HLA-I allotypes, higher *TAPBP* mRNA expression levels, as imputed based on genotypes of these two SNPs, associated with both decreased prevalence of *Plasmodium falciparum* (*P. falciparum*) parasites and decreased incidence of clinical malaria compared to subjects with lower imputed *TAPBP* mRNA expression levels. Among subjects with tapasin-independent HLA-I allotypes, there was little to no impact of differential imputed expression (hereafter referred to as “i-expression”) levels of *TAPBP* mRNA, and this group, as a whole, showed equivalent protection to that observed in the tapasin-dependent group with high *TAPBP* mRNA i-expression. These data suggest that the restrictive nature of tapasin dependence in terms of antigen presentation may be alleviated by higher *TAPBP* mRNA expression levels and emphasize the delicate balance of factors involved in antigenic peptide loading and presentation on the cell surface, affecting the course of human disease.

## Results

### SNPs in the 5′ UTR and 3′ UTR of *TAPBP* Determine Expression Levels of *TAPBP* mRNA.

**Fig. 1.**
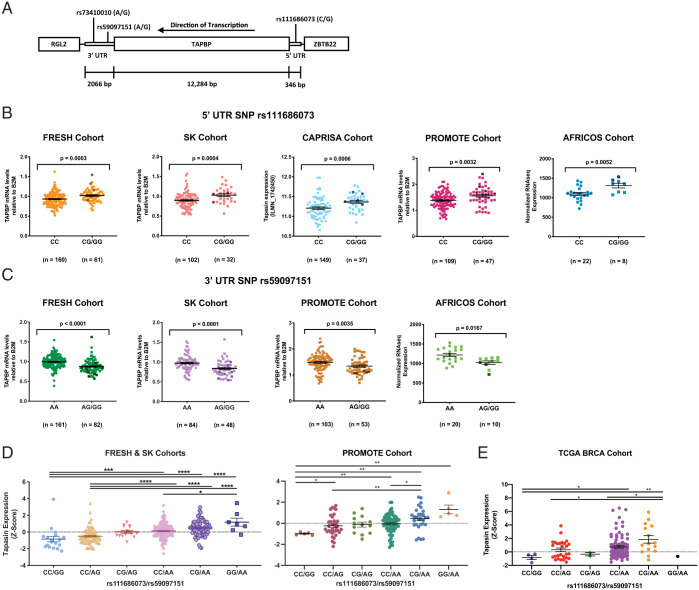
*rs111686073* and r*s59097151* genotypes associate with *TAPBP* mRNA expression level in PBMCs. (*A*) Schematic representation of *TAPBP* and adjacent genes roughly to scale, with relevant SNPs and the direction of transcription indicated. (*B* and *C*) *TAPBP* mRNA expression level was measured by using qPCR (FRESH and SK) and microarray data (CAPRISA) in each of three (for *rs111686073*) or two (for *rs59097151*) Black South African cohorts, and for the Ugandan cohorts, mRNA expression was measured by using qPCR in PROMOTE and RNA-Seq in AFRICOS: *rs111686073* in the FRESH (*n* = 221), Sinikithemba (*n* = 134), CAPRISA (*n* = 186), PROMOTE (*n* = 156), and AFRICOS (*n* = 30) cohorts (*B*); and *rs59097151* in the FRESH (*n* = 243), Sinikithemba (*n* = 132), PROMOTE (*n* = 156), and AFRICOS (*n* = 30) cohorts (*C*). Black symbols in the combined groups indicate homozygotes. (*D*) Expression across the combined genotypes of *rs111686073* and *rs59097151* in the pooled FRESH and SK cohorts (*n* = 384) and in the PROMOTE cohort (*n* = 156). mRNA levels were converted to Z-scores to allow for the combination of datasets in the case of the FRESH and SK cohorts. The numbers of individuals with each genotype are as follows: for the FRESH and SK cohort: *n*_CC/GG_ = 18, *n*_CC/AG_ = 90, *n*_CG/AG_ = 15, *n*_CC/AA_ = 186, *n*_CG/AA_ = 68, and *n*_GG/AA_ = 7; and for the PROMOTE cohort: *n*_CC/GG_ = 5, *n*_CC/AG_ = 35, *n*_CG/AG_ = 13, *n*_CC/AA_ = 69, *n*_CG/AA_ = 29, and *n*_GG/AA_ = 5. (*E*) *TAPBP* mRNA expression within tumors from African-American individuals in the TCGA breast cancer cohort (BRCA; *n* = 137). Expression is presented as Z-score normalization to the grand mean mRNA expression of all TCGA tumors. The numbers of individuals with each genotype for the TCGA cohort are as follows: *n*_CC/GG_ = 4, *n*_CC/AG_ = 27, *n*_CG/AG_ = 2, *n*_CC/AA_ = 88, *n*_CG/AA_ = 15, and *n*_GG/AA_ = 1. Mean ± SEM are shown, and significance was determined by using unpaired, two-sided Mann–Whitney tests. *P < 0.05; **P < 0.01; ***P < 0.001; ****P < 0.0001. B2M, *β2M*.

Expression databases have shown that *TAPBP* mRNA is expressed at varying levels among individuals (https://gtexportal.org/home/gene/TAPBP). We scanned the *TAPBP* gene for common SNPs and identified two that associated significantly with *TAPBP* mRNA expression levels, *rs111686073* (chr6:33314158 GRCh38.p13) and *rs59097151* (chr6:33301434 GRCh38.p13), located in the 5′ UTR and the 3′ UTR, respectively (Fig. 1*A*). A third SNP, *rs73410010* (chr6:33300359 GRCh38.p13), is in complete linkage disequilibrium (LD) with *rs59097151* (we refer only to *rs59097151* in the remainder of this section). The three SNPs are present in Black populations, but these positions are fixed in those of European descent. The minor *rs111686073G* variant was significantly associated with higher *TAPBP* mRNA expression in peripheral blood mononuclear cells (PBMCs) from three separate Black South African cohorts (Females Rising through Education, Support, and Health [FRESH], Sinikithemba [SK], and Centre for the AIDS Program of Research in South Africa [CAPRISA]; dominant model *P* = 0.0003, 0.0004, and 0.0006, respectively; Fig. 1*B*) and two separate Ugandan cohorts (the US President’s Emergency Plan for AIDS Relief Promoting Maternal and Infant Survival Everywhere Ongoing Treatment Evaluation [PROMOTE] and the African Cohort Study [AFRICOS]; dominant model *P* = 0.0032 and 0.0052, respectively; Fig. 1*B*). The minor *rs59097151G* variant was significantly associated with lower expression in the FRESH and SK cohorts (dominant model *P* < 0.0001 for each group; Fig. 1*C*; CAPRISA samples were not available for analysis), and the PROMOTE and AFRICOS cohorts (dominant model *P* = 0.0035 and 0.0167, respectively; Fig. 1*C*). *rs111686073* and *rs59097151* form three haplotypes with poor LD overall (D′ = 1, *r*^2^ < 0.05; *SI Appendix*, Fig. S1), indicating that the associations with expression are largely independent. A stepwise increase in *TAPBP* mRNA expression was observed in both the combined FRESH and SK cohorts and the PROMOTE cohort when ordering the compound genotypes of *rs111686073* and *rs59097151* by increasing numbers of high-expression alleles, with *rs59097151* appearing to have a slightly larger effect upon expression than *rs111686073* (Fig. 1*D*). A similar pattern of stepwise increase of *TAPBP* mRNA expression in accordance with the increasing number of high-expression alleles was observed in The Cancer Genome Atlas Project (TCGA) cohort for breast invasive carcinoma (BRCA; Fig. 1*E*), confirming the association of these two SNPs with *TAPBP* mRNA expression levels. We further observed significantly higher tapasin protein expression levels in B lymphoblastoid cell lines (BLCLs) from individuals with at least one *rs111686073G* variant (i.e., *CG* or *GG*), along with the *rs59097151AA* genotype, as compared to those with the *rs111686073CC/rs59097151GG* genotype (*P* = 0.0243; *SI Appendix*, Fig. S2).

### *rs111686073G* Confers Higher *TAPBP* mRNA Expression due to its Greater Binding Affinity for AP-2α.

**Fig. 2.**
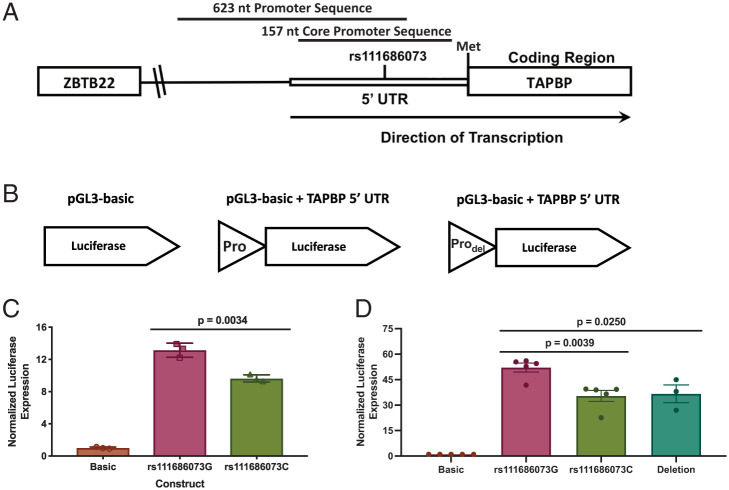
r*s111686073G* induces higher luciferase expression than the *C* variant. The pGL3-based luciferase constructs with a 623-nt promoter sequence containing the core promoter and SNP of interest was constructed by using Gibson assembly and transfected into HeLa cells for the initial analysis of the effect of *rs111686073* on *TAPBP* mRNA expression. To examine the effects of r*s111686073* more accurately, a shorter, 157-nt promoter sequence containing the proximal promoter and either the SNP of interest or a 10-nt deletion was constructed by using Gibson assembly and transfected into MCF7 cells. Luciferase activity was measured 48 h posttransfection and normalized to the expression of Renilla and the empty pGL3-basic vector (Promega). (*A*) Map of the area around *rs111686073* with the promoter sequences for the luciferase assay indicated (not to scale). (*B*) Schematics of the pGL3-based luciferase constructs, with “Pro” indicating the location of the promoter insert (i.e., *TAPBP* 5′ UTR) containing *rs111686073* and “Pro_del_” indicating the promoter with the 10-nt deletion. (*C*) The 623-nt construct containing the *G* variant shows significantly higher luciferase expression, as compared to that with the *C* variant. (*D*) The 157-nt construct containing the *G* variant shows significantly higher luciferase expression, as compared to the *C* variant, or a 10-nt deletion that deletes the AP-2α binding site. The mean ± SEM of at least three independent experiments is shown, and significance was determined by using unpaired, two-sided Student’s *t* tests.

**Fig. 3.**
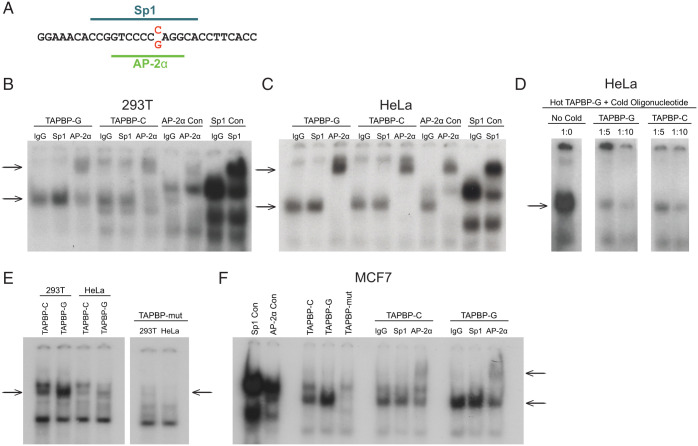
AP-2α binds r*s111686073G* more strongly than *rs111686073C*. EMSAs were performed with oligonucleotides containing the two respective variants of the SNP and consensus sequences for Sp1 and AP-2α. (*A*) Web-tool Alibaba2 predicted Sp1 and AP-2α binding sites in the 5′ UTR of *TAPBP* overlapping *rs111686073*. The SNP variants are indicated in red, and the lines indicate the predicted binding sites. (*B* and *C*) Nuclear extract from 293T cells (*B*) and HeLa cells (*C*) bound to both the *C*- and *G*-variant oligomers (lower arrows), and shifts were observed in the presence of antibody to AP-2α (higher arrows), but not in the presence of the control IgG or antibody to Sp1. Binding of AP-2α to the *TAPBP-G* oligomer appears stronger than binding to the *TAPBP-C* oligomer. Positive control Sp1 and AP-2α consensus binding-site oligomers (Sp1 Con and AP-2α Con, respectively) show protein shifts with antibodies to Sp1 and AP-2α, respectively. (*D*) A cold competition assay was performed with HeLa nuclear extract using 1:0, 1:5, and 1:10 ratios of hot to cold oligomer run on a single gel. Cold *G*-variant oligomer was better able to compete with hot *G*-variant oligomer and resulted in fainter bands relative to the cold *C*-variant oligomer. (*E*) Binding of nuclear extracts from 293T and HeLa cells is abrogated when the overlapping AP-2α and AP-2γ sites are mutated (*TAPBP*-mut oligomer). Arrows indicate location for the band associated with AP-2α binding. (*F*) Nuclear extract from MCF7 cells also shows abrogation of binding to the *TAPBP*-mut oligomer. AP-2α binds both the *TAPBP-C* and *TAPBP-G* oligomers (lower arrow), as indicated by the shifts in the presence of antibody to AP-2α (higher arrow).

The potential direct effect of *rs111686073* (*C*/*G*) on *TAPBP* mRNA expression was examined by cloning the respective alleles of *rs111686073* within the 5′ UTR of *TAPBP* along with the core promoter (623 bp total, differing only at *rs111686073*) into the luciferase expression vector pGL3 (Fig. 2 *A* and *B*). Luciferase expression was significantly higher in HeLa cells transfected with the *rs111686073G* variant than the *rs111686073C* variant (*P* = 0.0034; Fig. 2*C*), indicating stronger transcriptional activity of the insert containing *rs111686073G*. The web tool Alibaba2 predicted that transcription factors Sp1 and AP-2α bound sequences overlapping *rs111686073* (Fig. 3*A*). Using an electrophoretic mobility shift assay (EMSA), we observed binding of AP-2α to oligomer probes containing the *G* or *C* variants in HeLa and 293T cells, while Sp1 did not bind to either probe in these cell lines (Fig. 3 *B* and *C*). The *G* variant showed greater binding affinity for AP-2α than did the *C* variant at *rs111686073*, which was most apparent in the HeLa cell extract, perhaps due to the higher amount of AP-2α protein observed in the HeLa extract compared to that in the 293T cell extract.

The differential AP-2α binding affinity for the two *rs111686073* variants was verified by using a cold competition assay with radioactive *G*-variant oligonucleotides titrated with cold *G*- or *C*-variant oligonucleotides using HeLa cell extracts (Fig. 3*D*). Competition with either of the cold probes at 5-fold or 10-fold greater concentrations than the hot probe resulted in decreased binding of the respective hot oligonucleotides, but the cold probe containing the *G* variant competed more effectively than did the cold probe containing the *C* variant (Fig. 3*D*). Thus, AP-2α has a stronger affinity for the binding site containing *rs111686073G*, as compared to *rs111686073C*, corresponding to the enhanced transcriptional activity of luciferase in the presence of *rs111686073G* relative to *rs111686073C*. To further verify that the differential binding of AP-2α is influencing the expression of *TAPBP* and to reduce interference from alternative upstream promoters present in the larger fragment that was initially used, we cloned into the luciferase expression vector an insert containing only the proximal promoter (157 bp total) that included either of the *rs111683073* variants (*G* or *C*) or a 10-bp deletion that eliminated the AP-2α binding site completely (Fig. 2*A* and *B*). The luciferase assay with the proximal promoter was carried out in the breast cancer cell line MCF7, which is known to have high AP-2α and AP-2γ expression (19). The *rs111686073G* construct induced higher expression of luciferase than either the *rs111686073C* or the deletion constructs in these cells (Fig. 2*D*). Furthermore, in an EMSA, mutating the AP-2α binding site resulted in loss of protein binding to the oligomer probe in 293T and HeLa cell extracts (Fig. 3*E*), as well as MCF7 cell extract (Fig. 3*F*). Overall, these data strongly support a role for *rs111686073* in directly regulating *TAPBP* mRNA expression levels, where the higher affinity between AP-2α and *rs111686073G* accounts for the association between this variant and higher expression of *TAPBP* mRNA.

### Differential Binding of miR-4486 Is Responsible for the Effect of *rs59097151* on *TAPBP* mRNA Expression Levels.

**Fig. 4.**
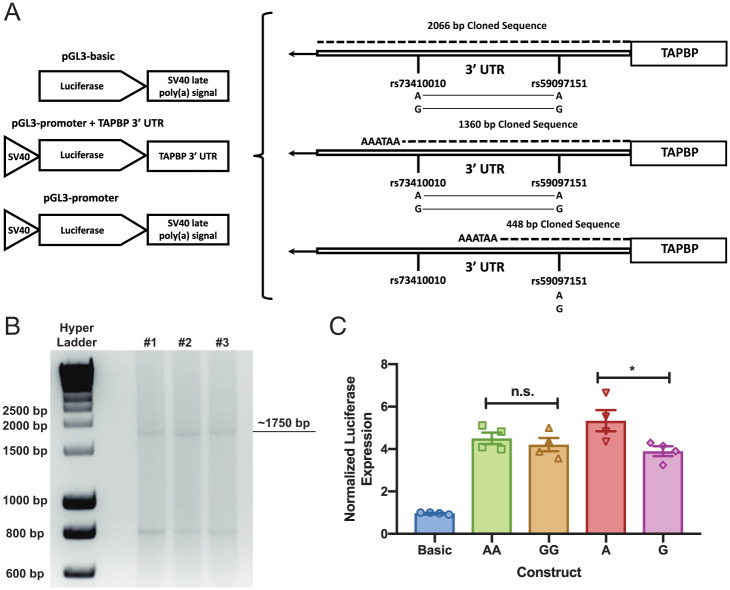
*rs59097151A* constructs express more luciferase than do *rs59097151G* constructs. pGL3-based luciferase constructs with abbreviated 3′vUTR sequences of *TAPBP* (1,360 bp and 448 bp) were constructed by using Gibson assembly and site-directed mutagenesis. The constructs were transfected into 293T cells, and luciferase activity was measured 48 h posttransfection. Luciferase activity was normalized to the expression of Renilla and the empty pGL3-basic vector (Promega). (*A*) Schematics for the various luciferase constructs and maps of the area around the SNPs (not to scale). Only variant combinations of *rs59097151* and *rs73410010* genotypes found in our African samples were examined. Haplotypes for the two long constructs are indicated by the connected nucleotides shown beneath the two SNPs, and the variants for the shortest construct (448 bp) by the nucleotides below the included SNP (i.e., *rs59097151*). (*B*) Nested PCR of 3′ RACE confirmed the length of the full 3′vUTR of *TAPBP* with RNA isolated from PBMCs of three donors. Bands indicated were positively identified as the *TAPBP* 3′vUTR via Sanger sequencing. (*C*) The 293T luciferase experiments with constructs containing the two 3′vUTR haplotypes described in *A* or the two variants of *rs59097151* (448 bp). The haplotypes of the 1,360-bp constructs are indicated by *A*/*A* and *G*/*G* and the alleles at *rs59097151* of the 448-bp constructs by *A* and *G*. The mean ± SEM of four independent experiments is shown, and significance was determined by using unpaired, two-sided Student’s *t* tests. *P < 0.05. n.s., not significant.

The 3′ UTR of *TAPBP* is 2,066 bp long (Fig. 4*A*) and fully transcribed (a nested 3′ Rapid Amplification of cDNA Ends [RACE] of the full 2,066 bp results in a 1,750-bp product; Fig. 4*B*). As *rs73410010* and *rs59097151* are located only 1,074 bp apart within the 3′ UTR and are in perfect LD with one another, either could be directly responsible for their association with *TAPBP* mRNA expression levels. In order to address this, three luciferase constructs were tested (Fig. 4*A*). An insert of the full 2,066-bp 3′ UTR sequence containing either *rs73410010A/rs59097151A* (*A*/*A*) or *rs73410010G/rs59097151G* (*G*/*G*) resulted in minimal luciferase expression, regardless of genotype or cell type used in the assay (HeLa, 293T, and Jurkat cell lines; 293T cells shown in *SI Appendix*, Fig. S3). This limited luciferase expression may be due to the close proximity of the *TAPBP* and *RGL2* genes, which are transcribed in the same direction with only 245 bp separating the polyA site of *TAPBP* and the 5′-most transcription start site of *RGL2*. Transcription-factor binding elements that regulate *RGL2* are likely present in the 3′ region of the *TAPBP* 3′ UTR. The terminal 625 bp of the *TAPBP* transcript is relatively GC-rich (59.2% GC) and contains multiple Sp1-binding elements, which could recruit RNA polymerase II and compete with the SV40 promoter driving luciferase expression from the reporter vector. Furthermore, if these elements generate antisense transcripts, they could lead to the degradation of luciferase transcripts. In order to prevent any potential interference, a shorter insert was generated containing only the first 1,360 bp of the 3′ UTR and a synthetic poly-adenylation site (AATAAA) (Fig. 4*A*). The 293T cells transfected with constructs containing either the *A*/*A* or *G*/*G* 1,360-bp insert showed no significant difference in luciferase signal (*P* = 0.06; Fig. 4*C* and *SI Appendix*, Fig. S2). An even shorter insert containing only the first 448 bp (thus excluding *rs73410010*; Fig. 4*A*) showed that the *rs59097151A* variant induced significantly higher luciferase expression than did the *G* variant (*P* = 0.04; Fig. 4*C* and *SI Appendix*, Fig. S3), implicating *rs59097151* as the SNP directly responsible for the observed differential *TAPBP* mRNA expression.

**Fig. 5.**
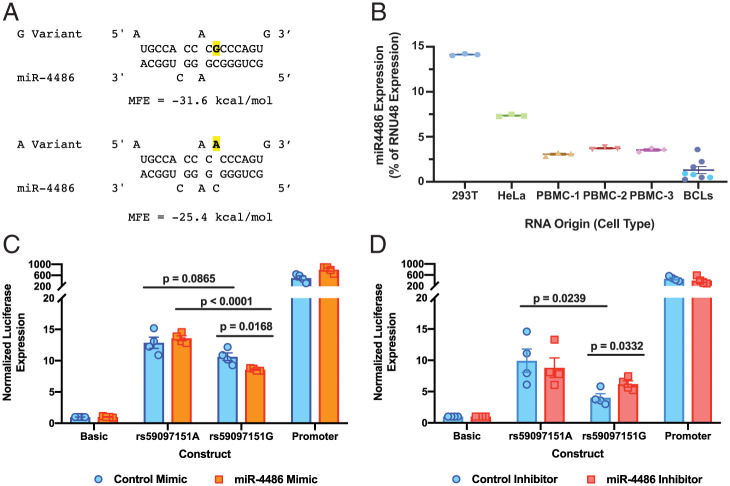
miR-4486 binds *rs59097151G* and results in decreased luciferase expression. (*A*) RNAhybrid 2.2 predictions for miRNA binding and the minimum free energy (MFE) of the duplexes are shown. The nucleotide variant of *rs59097151* is indicated in bold and highlighted. (*B*) Expression of mature miR-4486 as a percentage of RNU48 (a small nucleolar RNA) expression in 293T cells, HeLa cells, and PBMCs from three individuals and B-cell lines derived from eight individuals. miR-4486 expression was quantitated by using the 2^-ΔΔCt^ method with miR-423 as the endogenous control and RNU48 as the reference for expression. In the B-cell-line category, the turquoise symbols indicate individuals homozygous for *rs59097151G* (*n* = 3), and the remaining in dark blue (*n* = 5) are composed of two heterozygotes and three *rs59097151A* homozygotes. Samples were measured by using TaqMan miR qPCR assays in triplicate. (*C* and *D*) The 448-bp *TAPBP* 3′ UTR luciferase constructs containing only the *rs59097151* variants were transfected into 293T cells with Renilla and either an miR-4486 mimic and control miRNA mimic (*C*) or an miR-4486 inhibitor and control miRNA inhibitor (*D*), as indicated. After 2 d, cells were lysed, and luciferase activity was read and normalized to the expression of Renilla and the empty pGL3-basic vector. The pGL3-Promoter construct expression is included as a positive control. The mean ± SEM of four independent experiments is shown, and significance was determined by using unpaired, two-sided Student’s *t* tests. Only the *G* variant is susceptible to down-regulation by miR-4486 mimic (*C*), while there is significantly increased expression for the *G* variant in the presence of the miRNA inhibitor (*D*).

In silico prediction of miR binding sites was performed by using miRDB (Version [v] 5.0; mirdb.org) (20, 21), which implicated miR-4486 in the binding sequence containing *rs59097151G* (target score = 84), but not *rs59097151A*. TargetScan (Release 7.2; https://www.targetscan.org/vert_72/) (22) confirmed the likelihood of miR-4486 binding to the same site when the *G* variant, but not the *A* variant, is present. Alignment of mature miR-4486 to the 3′ UTR using RNAhybrid (v2.2) (23) further indicated that the binding of miR-4486 is more stable when the *G* variant is present, as indicated by a lower free-energy requirement for binding and a longer uninterrupted seed region than when the *A* variant is present (Fig. 5*A*). The 293T cells, HeLa cells, and PBMCs from three individuals were examined by qPCR for expression of mature miR-4486, as well as two highly and consistently expressed small RNAs, miR423 (selected as an endogenous control) and RNU48 (a small nucleolar RNA widely expressed across tissue types). The miR423 and RNU48 RNAs were detected in all cell types at levels above the negative control (i.e., no reverse transcriptase) (*SI Appendix*, Fig. S4*A*). Expression of miR-4486 was then analyzed via the 2^-ΔΔCt^ method relative to the expression of RNU48 (Fig. 5*B*). All cell types tested expressed miR-4486 (Fig. 5*B*), in line with the ubiquitous expression of miR-4486 reported in the Tissue Atlas database [https://ccb-web.cs.uni-saarland.de/tissueatlas/ (24); *SI Appendix*, Fig. S4*B*].

Cotransfection of either an miR-4486 mimic or inhibitor into 293T cells along with the *TAPBP* 3′ UTR luciferase constructs containing only the respective *rs59097151* variants (448-bp construct) was performed to validate the specificity of miR-4486 in regulating luciferase expression through the 3′ UTR of *TAPBP*. Nonspecific mimic and inhibitor sourced from *Caenorhabditis elegans* with minimal sequence identity in humans were used as controls. Cotransfection of the expression construct containing *rs59097151G* and miR-4486 mimic induced a significant decrease (0.807-fold) in luciferase expression compared to that with control mimic (*P* = 0.017); however, there was no effect of miR-4486 mimic on luciferase expression for the *rs59097151A* construct (Fig. 5*C*). In general, even in the presence of control mimic, the construct containing *G* produced lower luciferase expression compared to the construct containing *A*, which is likely attributable to an effect of endogenous miR-4486 on the expression construct containing *G*. Cotransfection of the expression construct containing *rs59097151G* and the miR-4486 inhibitor induced a significant increase (1.54-fold) in luciferase expression, compared to that with control mimic (*P* = 0.033), but there was no significant effect of miR-4486 inhibitor on luciferase expression for the *A*-variant construct (Fig. 5*D*). Once again, the construct containing *G* produced lower luciferase expression compared to the construct containing *A*, even in the presence of control inhibitor. Together, the effect of miR-4486 mimic and inhibitor suggest that this miR is directly responsible for the association between *TAPBP* mRNA expression levels and the *rs59097151* genotype.

### Higher *TAPBP* mRNA i-Expression Levels Are Associated with Lower Parasite Prevalence in *P. falciparum* Infection.

We next sought to determine whether *TAPBP* mRNA expression levels are associated with blood-stage *P. falciparum* infection and/or clinical malaria among Ugandan individuals enrolled in a large malaria cohort study. Characteristics of the study population were previously reported in Digitale et al. (25) (see *Clinical and Parasitological Outcomes of the Malaria Cohort* for a brief overview). The study was conducted at three sites with differing levels of malaria-transmission intensity (26); malaria incidence was highest in Tororo, intermediate in Kanugu, and lowest in Jinja, reflecting the varying levels of exposure to infected mosquitos across these sites (27). Four outcomes were considered: the prevalence of *P. falciparum* parasitemia (per quarter); the incidence of clinical malaria, defined as parasitemia plus fever or other symptoms (number of malaria episodes per person-year); parasite density at routine quarterly visits; and parasite density at symptomatic malaria visits. Parasite density (either during routine or malaria visits), a measure of blood-stage control of the parasite involving antibodies, as opposed to cellular immune mechanisms, showed no significant effects after correction for multiple comparisons in any of our analyses and was not considered further. All multivariable models included adjustment for the following covariates: study site, age, sex, and household mosquito-capture data (calculated as the entomological inoculation rate, or EIR). The relationship of these covariates to malaria outcome measures is shown in *SI Appendix*, Table S1.

**Fig. 6.**
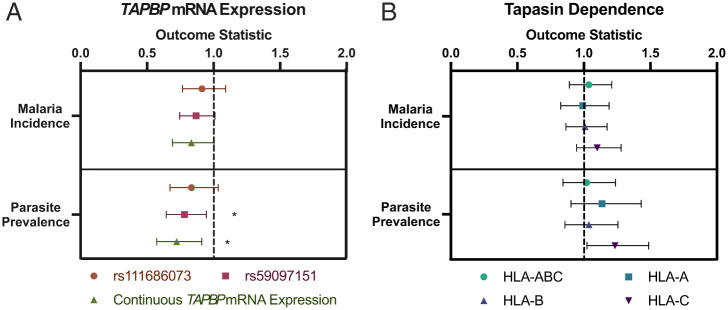
Higher *TAPBP* mRNA i-expression associates with lower parasite prevalence. (*A*) The two malaria outcomes were examined for an association with *TAPBP* mRNA i-expression based on *rs111686073* genotype (*GG*/*CG* vs. *CC*) or *rs59097151* genotype (*AA* vs. *AG*/*GG*), each as a dichotomous variable and as a combined continuous variable (continuous *TAPBP* mRNA i-expression) with three levels of mRNA i-expression, based on data in Fig. 1*D* (denoting *rs111686073*/*rs59097151* genotypes, respectively): *CC*/*GG* and *CC*/*AG* (lowest); *CG*/*AG* and *CC*/*AA* (intermediate); and *CG*/*AA* and *GG*/*AA* (highest). (*B*) Associations between malaria outcomes and tapasin dependence as a dichotomous variable are shown for the combined HLA-A, -B, and -C allotypes and each locus individually. Empirically defined cutoffs (HLA-ABC: 1.7, HLA-A: 1.1, HLA-B: 1.4, and HLA-C: 0.8) were used to distinguish tapasin dependence vs. independence (*Materials and Methods*). Malaria incidence = number of symptomatic malaria episodes per person-year (IRR ± bounds); parasite prevalence = having at least one symptomatic or asymptomatic parasitemic visit per quarter (OR ± bounds). FDR q-values are displayed. *q < 0.05.

*TAPBP* mRNA i-expression levels were analyzed by using *rs111686073* as a dichotomous variable (*CG*/*GG* [high expression] vs. *CC* [low expression]), *rs59097151* as a dichotomous variable (*AA* [high expression] vs. *AG*/*GG* [low expression]), and expression as a continuous variable based on three groupings of SNP genotypes derived from Fig. 1*D* (denoting *rs111686073*/*rs59097151* genotypes, respectively): *CC*/*GG* and *CC*/*AG* (lowest); *CG*/*AG* and *CC*/*AA* (intermediate); and *CG*/*AA* and *GG*/*AA* (highest). Three groupings were used for analysis due to the very close mean expression values for each of the two lowest-, two intermediate-, and two highest-expression genotypes (Fig. 1*D*). The distribution of genotypes and i-expression groupings is shown in *SI Appendix*, Table S2. SNP genotype frequency varied somewhat by site, but did not correspond to the hierarchy of malaria incidence or EIR (e.g., Jinja, the lowest malaria incidence site, was intermediate for frequency of both SNPs). Parasite prevalence and malaria incidence showed significant associations with TAPBP mRNA i-expression level, where higher i-expression level conferred protection for both outcomes using the *rs59097151* genotype alone (malaria incidence: incidence rate ratio [IRR] = 0.868, false discovery rate [FDR] q-value = 0.159; parasite prevalence: odds ratio [OR] = 0.781, FDR q-value = 0.027) and the combined *rs111686073*/*rs59097151* genotypes (malaria incidence: IRR = 0.833, FDR q-value = 0.110; parasite prevalence: OR = 0.722, FDR q-value = 0.011; Fig. 6*A* and *SI Appendix*, Table S3). Though not statistically significant, higher *TAPBP* mRNA i-expression based on the *rs111686073* genotype also trended toward protection against both outcomes (Fig. 6*A* and *SI Appendix*, Table S3).

### Higher *TAPBP* mRNA i-Expression Is Associated with Protection among Subjects with Tapasin-Dependent Allotypes.

The intrinsic variation of HLA-I allotypes in their dependence upon tapasin has been shown to impact the breadth of peptides presented to T cells (11). We previously determined tapasin dependence values for 97 HLA-I allotypes (11), 83 of which were observed in the Ugandan cohorts. An additional 34 allotypes were present in our Ugandan cohorts, so tapasin-dependence values for these allotypes were determined (total *n* = 117; *SI Appendix*, Fig. S5). Tapasin dependence was analyzed as a dichotomous variable with empirically determined groupings of tapasin-dependent or -independent allotypes of HLA-A, -B, and -C individually and HLA-A/-B/-C combined (i.e., the combination of the six values representing each allotype of HLA-A, -B, and -C in an individual) (11) (*SI Appendix*, Fig. S6). Higher HLA-C tapasin dependence associated weakly with parasite prevalence (OR = 1.234, FDR q-value = 0.053; Fig. 6*B* and *SI Appendix*, Table S4), but, overall, the level of tapasin dependence on its own had little to no effect on malaria outcomes.

**Fig. 7.**
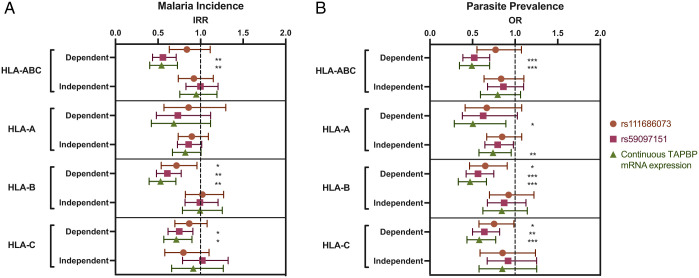
Higher *TAPBP* mRNA i-expression associates with protection against malaria outcomes among subjects with tapasin-dependent HLA-I allotypes specifically. Expression was examined as either a dichotomous variable based on *rs111686073* (*GG*/*CG* vs. *CC*) or *rs59097151* genotypes (*AA* vs. *AG*/*GG*) or as a continuous variable with three groups of expression levels (as described in the Fig. 6 legend). Empirically defined cutoffs (HLA-ABC: 1.7, HLA-A: 1.1, HLA-B: 1.4, and HLA-C: 0.8) were used to distinguish tapasin-dependent vs. -independent levels (*Materials and Methods*). Among subjects with tapasin-dependent HLA-I allotypes, high *TAPBP* mRNA i-expression levels associate with lower malaria incidence (*A*; number of symptomatic malaria episodes per person-year) and lower parasite prevalence (*B*; defined as having at least one symptomatic or asymptomatic parasitemic visit per quarter), compared to those with low *TAPBP* mRNA i-expression levels. Weaker to no effect of *TAPBP* mRNA i-expression levels were observed among those with tapasin-independent allotypes. See *SI Appendix*, Table S5 for numbers in each subgroup (total *n* = 833 for malaria incidence, total *n* = 835 for parasite prevalence). IRR: upper and lower bounds are displayed. FDR q-values are displayed. *q < 0.05; **q < 0.01; ***q < 0.001.

Variation in *TAPBP* mRNA i-expression levels would be predicted to have a greater impact on HLA-I allotypes that are highly dependent on tapasin for peptide loading relative to those that can load peptide even in the absence of tapasin. We stratified subjects into two groups, those with tapasin-dependent genotypes and those with tapasin-independent genotypes, based on allotypic tapasin-dependence values determined for HLA-A, -B, and -C individually and combined HLA-A/-B/-C (dichotomous divisions shown in *SI Appendix*, Fig. S6) and tested for a potential effect of *TAPBP* mRNA i-expression level on disease outcome in each grouping separately. High *TAPBP* mRNA i-expression levels, particularly based on the *rs59097151* genotype alone or the combined *rs111686073*/*rs59097151* genotype, associated with protection against both malaria incidence and parasite prevalence most strongly and consistently within the tapasin-dependent allotype groupings (Fig. 7 and *SI Appendix*, Table S5). High *TAPBP* mRNA i-expression level based on *rs11686073* alone was also associated with significant protection in the HLA-B tapasin-dependent grouping. Overall, the impact of *TAPBP* mRNA i-expression levels as a function of tapasin-dependence grouping was greatest for HLA-B relative to HLA-A and -C. The tapasin-dependent HLA-A grouping showed stronger protection with higher *TAPBP* mRNA i-expression levels than did the tapasin-independent grouping (based on IRR and OR), but significant associations occurred more frequently in the tapasin-independent group; this is likely due to greater statistical power in the tapasin-independent HLA-A group (*n* = 690), compared to the tapasin-dependent HLA-A group (*n* = 143; *SI Appendix*, Fig. S6). Overall, higher *TAPBP* mRNA i-expression levels associate with protection against malaria incidence and parasite prevalence among individuals with tapasin-dependent HLA-I allotypes, while there is no substantial benefit for those with tapasin-independent allotypes.

**Fig. 8.**
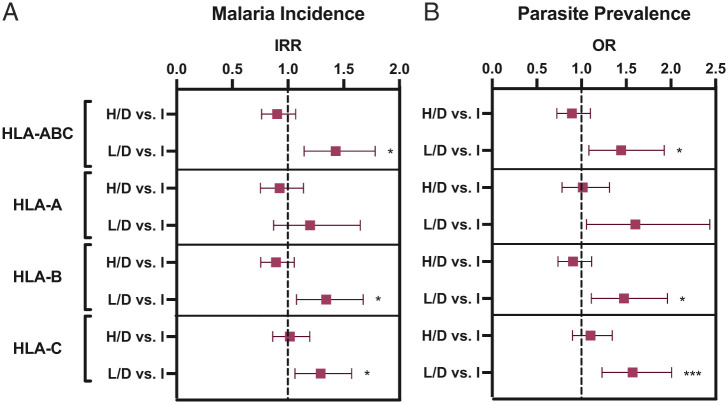
High *TAPBP* mRNA i-expression levels in individuals with tapasin-dependent HLA-I allotypes confer equal protection to that observed among all individuals with tapasin-independent allotypes. Individuals with tapasin-dependent allotypes who have either high or low *TAPBP* mRNA i-expression levels, as measured by *rs59097151* as a dichotomous variable (*AA* vs. *AG*/*GG*), were compared to all individuals with tapasin-independent HLA allotypes to determine their relative effects on malaria outcomes. Empirically defined cutoffs (HLA-ABC: 1.7, HLA-A: 1.1, HLA-B: 1.4, and HLA-C: 0.8) were used to distinguish tapasin-dependent vs. independent levels (*Materials and Methods*). Individuals with tapasin-dependent allotypes who had high i-expression levels of *TAPBP* mRNA showed equivalent protection to those with tapasin-independent allotypes against malaria incidence (*A*) and against parasite prevalence (*B*). H/D, high *TAPBP* mRNA i-expression and tapasin-dependent allotypes; L/D, low *TAPBP* mRNA i-expression and tapasin-dependent allotypes; I, tapasin-independent allotypes. Malaria incidence, number of symptomatic malaria episodes per person-year; parasite prevalence, having at least one symptomatic or asymptomatic parasitemic visit per quarter. IRR: upper and lower bounds are displayed. FDR q-values are displayed. *q < 0.05; ***q < 0.001.

Individuals in the tapasin-dependent groups who had high *TAPBP* mRNA expression, as inferred by the *rs59097151* genotype, showed a similar level of protection as individuals with tapasin-independent allotypes (regardless of *TAPBP* mRNA i-expression) against malaria incidence and parasite prevalence (Fig. 8 and *SI Appendix*, Table S6). The combination of low *TAPBP* mRNA i-expression with tapasin-dependent HLA-I allotypes significantly correlated with increased risk of malaria incidence and parasite prevalence, in comparison to those with tapasin-independent allotypes (Fig. 8). Together, the data indicate that tapasin dependence of HLA-I allotypes is a risk factor for malaria outcomes when coupled with low *TAPBP* mRNA i-expression and that high *TAPBP* mRNA i-expression among those with tapasin-dependent allotypes correlates with equivalent protection to that of tapasin-independent allotypes overall.

## Discussion

Tapasin is an integral part of the PLC and facilitates MHC-I–mediated antigen presentation. Down-regulation of tapasin is an established mechanism for immune evasion, and, here, we identified two SNPs, *rs111686073* and *rs59097151*, in the *TAPBP* gene found only in Black populations that significantly associate with *TAPBP* mRNA expression in PBMCs. The 5′ UTR SNP *rs111686073* regulates *TAPBP* mRNA expression through differential binding affinity of the transcription factor AP-2α, which has increased affinity for the minor variant (*G*), resulting in higher *TAPBP* mRNA expression. The second variant, *rs59097151*, is located in the 3′ UTR of the gene and regulates *TAPBP* mRNA expression through differential binding to miR-4486. The presence of the minor *G* variant allows miR-4486 binding, resulting in reduction of *TAPBP* mRNA expression, whereas the common *A* variant disrupts the miR-4486 binding site, resulting in higher expression of *TAPBP* mRNA. *AP-2α* mRNA and miR-4486 appear to be expressed ubiquitously across tissue types (GTEx and TissueAtlas), and their influence on *TAPBP* mRNA expression level is likely to dictate tapasin protein-expression level. Given the function of tapasin in stabilizing HLA-I in the absence of bound peptide, as well as editing peptides that bind in the groove of HLA-I, these two SNPs are likely to affect both the quality and breadth of the peptide-binding repertoire across cell types. It is very likely that other factors, such as epigenetic control or environmental effects, are involved in the regulation of tapasin expression at both the mRNA and protein levels, hence the variability in expression levels across individuals carrying the same genotype (Fig. 1). This is a limitation of our study, but it is similar to the variability seen in published studies examining correlations between expression quantitative trait loci and mRNA expression levels (28, 29). Importantly, we determined the regulatory basis for the differential expression across the genotypes of the two SNPs. It is likely that the variability in expression level across individuals carrying the same genotype (as shown in Fig. 1) does indeed add noise to our genetic associations with malaria outcomes, but, still, those genotype–disease associations are significant and support our biologically parsimonious model. Nevertheless, further in vitro assays to address functional and mechanistic pathways will be needed to confirm our findings. Previous studies have identified associations between variation in or near *TAPBP* with clearance of hepatitis C (*rs2071888*) (30) and survival in colorectal cancer (*rs3106189*) (31). While the mechanisms of these associations were not established, neither of these SNPs correlated with *TAPBP* mRNA expression level in our study, such that the influence of these variants on tapasin function could be distinct or depend on the cellular context.

HLA-I allotypes depend on tapasin to widely differing extents for peptide loading and surface expression (11–16), and it is likely that this variation in tapasin dependency affects the amount and quality of antigen presentation, as well as immunodominance (32, 33). Our analysis of *TAPBP* mRNA i-expression on malaria outcomes in Uganda took into account the level of tapasin dependence of HLA-I allotypes, and, as predicted, the *TAPBP* mRNA i-expression levels strongly modified the effect of tapasin-dependent allotypes on malaria outcomes, whereas they had little to no effect on tapasin-independent allotypes. Among those with tapasin-dependent allotypes, higher *TAPBP* i-expression levels (compared to lower) associated with protection against both parasitemia and clinical malaria to an extent that is equivalent to having tapasin-independent allotypes (regardless of *TAPBP* mRNA i-expression levels). Thus, the genotypes that are most restrictive in peptide-loading efficiency (i.e., those encoding tapasin-dependent allotypes and low levels of *TAPBP* mRNA i-expression) associate with poor control of *P. falciparum*. These data suggest that higher tapasin protein-expression levels in the ER may compensate for the apparent impediment of tapasin-dependent allotypes in this disease.

The immune response to malaria infection is complex, due to the intricate multistage development of *P. falciparum* in the vertebrate host, which includes initial asymptomatic infection of hepatocytes (liver stage) and subsequent infection of erythrocytes (blood stage), which is often accompanied by malaria symptoms (reviewed in ref. 34). T-cell responses have been shown to contribute immunity to both stages of *Plasmodium* infection, but are especially critical during the initial liver stage when the infectious inoculum is lowest. After exiting hepatocytes, *P. falciparum* establishes a self-propagating cycle of infection within erythrocytes, which lack HLA expression on the cell surface (34). Combinations of *TAPBP* mRNA i-expression and tapasin-dependence levels were associated with both blood-stage parasitemia and clinical malaria, both of which are influenced by liver-stage immunity, but these variants had no impact on parasite density, an outcome determined at the blood stage of disease. These data suggest that the strength of the CTL response in the liver, as determined in part by the efficacy of peptide loading of HLA-I molecules, influences the likelihood of *P. falciparum* infection progressing to the blood stage. Although sterilizing immunity against *P. falciparum* is rarely, if ever, fully achieved in response to natural infection, perhaps due to low antigen inoculum in the liver (35), liver-resident memory CTLs have been shown to be essential for protection against malaria during the liver stage in both mouse (36) and human (37) vaccine studies.

In all observational cohort studies, confounding is a potential concern. Parasite prevalence and malaria incidence are greatly influenced by factors such as age (due to the gradual acquisition of adaptive immunity) and the intensity of exposure to mosquitos harboring *P. falciparum* sporozoites, which can be remarkably heterogeneous within populations, even over small spatial scales (38). To control for this heterogeneity in exposure, we incorporated mosquito-capture measurements within each household to calculate a household-level EIR for all cohort participants. While increased age was indeed associated with protection from parasitemia and clinical malaria in the present cohort, the frequency of *rs111686073* and *rs59097151* did not differ by age strata in our cohort. The frequency of these genotypes did vary somewhat across the study sites, but showed no consistent relationship with malaria-exposure intensity. Further, we controlled for age, study site, and measured household log EIR in all models. Thus, it is unlikely that our results can be attributed to confounding by age or malaria-exposure intensity.

In conclusion, *rs111686073* and *rs59097151* in combination regulate *TAPBP* mRNA expression levels in Black populations. Among the more tapasin-dependent HLA-I allotypes, *TAPBP* mRNA i-expression levels associate with malaria disease outcomes, providing insight into the growing body of work on the genetic regulation of HLA-I–mediated antigen presentation and malaria-specific immune responses (25, 39). Further investigation into the concrete effects of *TAPBP* mRNA expression levels on the selection of epitopes by various HLA-I allotypes will help inform how immune responses are formed and immunodominance develops in healthy and pathogenic states. As *P. falciparum* is highly complex antigenically, it is likely that multiple epitopes are available for binding to and restriction by most HLA-I types, and our results suggest that it is the efficiency at which this process occurs that determines liver-stage control of this pathogen. It may be useful to consider these data in vaccine design against *P. falciparum* malaria.

## Materials and Methods

### Study Design.

Given the role of tapasin in shaping the HLA-I peptide repertoire and its impact on disease outcome, we hypothesized that natural variation in tapasin expression level could also affect disease pathogenesis and susceptibility. We identified two SNPs in Black populations that were found to associate significantly with *TAPBP* mRNA expression, as determined in three independent Black South African cohorts and two Ugandan cohorts. The mechanisms behind the two identified SNPs were elucidated through various assays. A published set of three parallel malaria cohorts in Uganda were then interrogated for the effect of *TAPBP* mRNA expression (as estimated by the SNP genotypes) on outcome after infection among subjects carrying HLA-I allotypes that depend on tapasin for peptide loading vs. those that do not depend on tapasin for peptide loading.

### Human Subjects.

Data generated in this study are from subjects enrolled in one of the following cohorts: SK, FRESH, CAPRISA, AFRICOS (40), PROMOTE (41), and three parallel cohort studies in Uganda conducted as part of the East Africa International Center of Excellence for Malaria Research (26). Blood samples for quantification of miRNA were acquired from the Research Donor Program of the National Cancer Institute (NCI) Frederick (NCI RDP). Normalized *TAPBP* mRNA expression was inferred from the BRCA cohort within TCGA(https://www.cancer.gov/about-nci/organization/ccg/research/structural-genomics/tcga). Ethical approval was granted by the Walter Reed Army Institute of Research Institutional Review Board; the Makerere University School of Public Health Institutional Review Board; the Makerere University School of Medicine Research and Ethics Committee; the London School of Hygiene and Tropical Medicine Ethics Committee; the University of California San Francisco Committee on Human Research; and the Uganda National Council for Science and Technology. All participants provided informed consent.

### Clinical and Parasitological Outcomes of the Malaria Cohort.

Data were collected from three parallel cohort studies in Ugandan sites with a range of malaria-transmission intensities conducted as part of the East Africa International Center of Excellence for Malaria Research. Nagongera, in Tororo district, is a rural area in southeastern Uganda with high transmission (parasite prevalence [% of quarters parasite positive] in children = 64.2). Kihihi, in Kanungu district, is a rural area in southwestern Uganda with moderate transmission (parasite prevalence in children = 36.4). Walukuba, in Jinja district, is a peri-urban area near Lake Victoria with relatively low transmission (parasite prevalence in children = 12.8). Approximately (approx.) 100 households were selected at each site, and all children aged 6 mo to 10 y (median age of 4.4 y, *n* = 187, Jinja; median age 4.7 y, *n* = 267, Kanungu; median age 5.1 y, *n* = 203, Tororo) and one adult caregiver were offered enrollment (*n* = 89, Jinja; *n* = 78, Kanungu; *n* = 66, Tororo). Participants were enrolled between August and October 2011 and followed through June 2016. In this analysis, visits after December 31, 2014, from Tororo were excluded because transmission changed dramatically at this site after intensive indoor residual spraying campaigns were started. Median analytic follow-up time was 55 mo in Jinja, 57 mo in Kanungu, and 39 mo in Tororo. More information on the cohorts is available from Kamya et al. (27) and Digitale et al. (25). Of 920 people in the cohorts, 835 people had complete genotype data for both *rs111686073* and *rs59097151*, and these were used in all analyses.

We collected outcome data via both active and passive surveillance. All participants visited the clinic quarterly for collection of thick blood smears and dried blood-spot samples. We asked caregivers to seek care for their children at the study clinic for any illness, free of charge. Children who were febrile (>38.0 °C) at the sick visit or who reported fever in the previous 24 h had thick blood smears obtained for microscopy. Malaria episodes, defined as parasitemia accompanied by self-reported or measured fever, were treated with artemether–lumefantrine per local guidelines if uncomplicated (42).

Entomological data were collected for each household monthly. We collected mosquitoes using miniature Centers for Disease Control and Prevention light traps and tested them for sporozoites by enzyme-linked immunosorbent assay (43). For each household, an annual EIR was calculated as the product of the yearly household human biting rate (geometric mean of female *Anopheles* mosquitoes caught in a household per day) and the site sporozoite rate (average proportion of mosquitos positive for *P. falciparum* at each site) (42).

### Quantification of *TAPBP* mRNA Expression.

*TAPBP* mRNA expression levels were measured in three South African cohorts, CAPRISA (HIV negative or preinfection; *n* = 186), FRESH (HIV negative, *n* = 243), and SK (treatment-naïve HIV-infected, *n* = 162); and two Ugandan cohorts, AFRICOS (*n* = 30) and PROMOTE (*n* = 156). RNA was extracted from PBMCs from the CAPRISA cohort by using TRIzol LS and treated with the RNA MinElute cleanup kit (Qiagen). The amount and integrity of the RNA was assessed by using a Bioanalyser RNA 6000 Nano kit (Agilent), and gene expression was quantified by using the Illumina HumanHT-12 v4 BeadChip gene-expression array platform with 47,231 probes, according to the manufacturer’s instructions. Total RNA from subjects in the FRESH and SK cohorts was extracted from PBMCs with the AllPrep DNA/RNA Mini Kit (Qiagen) and treated with DNase to remove genomic DNA. For the PROMOTE cohort, total RNA was extracted by using the RNeasy Plus Universal Mini Kit (Qiagen). After quantification via HT RNA Lab Chip (Caliper, Life Sciences), 1 µg of RNA was converted to complementary DNA (cDNA) via the High Capacity RNA-to-cDNA Kit (Applied Biosystems) for FRESH and SK and the SuperScript IV VILO kit with ezDNase enzyme (ThermoFisher Scientific) for PROMOTE. *TAPBP* and beta-2 microglobulin (*β2M*) mRNA were quantified by using the commercially available TaqMan Gene Expression Assay primer sets for each locus (Hs00175269_m1 and Hs00187842_m1, respectively) and the TaqMan Gene Expression Master Mix (ThermoFisher Scientific) on an ABI7900HT qPCR machine (FRESH and SK) or the QuantStudio 5 (PROMOTE). The average expression level of *TAPBP* mRNA was normalized to that of *β2M* RNA by using the 2^-ΔΔCt^ method. Total RNA from the AFRICOS participants was extracted from PBMCs by using the PureLink RNA Mini Kit (ThermoFisher Scientific), and sequence-ready libraries were constructed as described (44, 45). Libraries were sequenced on the Illumina NovaSeq 6000 by using an S1 Reagent Kit v1 (300 cycles) per the manufacturer’s instructions. Fastp v0.19.7 and Trimmomatic v0.33 with default parameters were used to trim low-quality bases from both ends of each read (46, 47). Trimmed reads were aligned to the human genome (GRCh38 build 88 to 92) by using HISAT2 v2.1.0 and counted with HTSeq (v0.6.1 to 0.9.1) (48, 49). The trimmed mean of M-values normalization method, as implemented in the R package edgeR, was used for normalization, and genotypes at SNP positions were determined by using the bcftools (v1.9) mpileup function with sorted binary alignment map files of RNA-sequencing (RNA-Seq) reads aligned to the human reference genome as input (50, 51).

### Tapasin Protein Western Blot.

Total protein was extracted by using radioimmunoprecipitation assay buffer from Epstein–Barr virus-transformed BLCLs (*n* = 19) grown to a density of 1 × 10^6^ cells/mL. Protein extracts were quantified by using the bicinchoninic acid assay, and 25 μg of each sample was loaded on NuPAGE 4 to 12% Bis-Tris gels (Thermofisher Scientific). Western blot analysis was performed according to standard manufacturer’s protocol using nitrocellulose membranes (Thermofisher Scientific). Ponceau staining was used to evaluate equal loading and transfer. The membranes were probed with anti-tapasin rat monoclonal antibody clone 7F6 (EMD Millipore) and goat anti-rat horseradish peroxidase-linked antibody (Cytiva). Signals were detected by using enhanced chemiluminescence reagent (Cytiva) and quantified by using GeneTools software (Syngene). Western blot experiments were repeated three times. For each experiment, protein samples from 19 BLCLs were quantified and loaded on three gels, so that each gel contained at least three samples that were also loaded on a different gel. These samples were used for signal normalization across the gels. Unpaired *t* tests were used to determine significance.

### Tapasin and *HLA* Genotyping.

*HLA* class I genotypes were determined by either the PCR–sequence-based typing protocol, as recommended by the 13th International Histocompatibility Workshop or by targeted next-generation sequencing, as described (11).

DNA from individuals in the three South African cohorts and the NCI RDP (the majority of the NCI RDP are of European descent) was sequenced for the 5′ UTR, exons, and 3′ UTR of *TAPBP*. Three gene segments were amplified with Platinum Taq DNA polymerase (Invitrogen) in several fragments: from the 5′ UTR to the beginning of the third intron (approx. 1.5 kb), from the end of the third intron to the beginning of the seventh intron (approx. 1.5 kb), and from the end of the seventh intron to the end of the 3′ UTR (approx. 2.1 kb) (see *SI Appendix*, Table S7, for primer sequences). The amplified fragments were sequenced by using the BigDye Terminator v1.1 Cycle Sequencing Kit (Applied Biosystems) and run on a 3730 Genetic Analyzer (Applied Biosystems) (primers used for sequencing are listed in the *SI Appendix*, Table S7). *rs111686073* and *rs59097151* were genotyped in the individuals from the Ugandan cohorts by amplifying the two regions containing the SNPs with Platinum Taq DNA polymerase (Invitrogen) (primers used for amplification and sequencing are listed in the *SI Appendix*, Table S7).

### Luciferase Reporter Construct Synthesis.

Two 623-bp fragments, differing only at *rs111686073* (G or C), of the *TAPBP* gene that contained the core promoter and a section of the 5′ UTR were amplified from genomic DNA (GRCh38.p13 position ch6:33314735 to ch6:33314114; please see the *SI Appendix*, Table S7, for all primers used in this section for construct synthesis and modification). Two smaller fragments of 157 bp, differing only at *rs111686073* (G or C), that contained only the proximal promoter were amplified from the 623-bp fragments (Chr38.p13 position ch6:33314220 to ch6:33314064). Nucleotides were added via PCR for insertion between XhoI (5′) and HindIII (3′) restriction sites for Gibson assembly with the pGL3-Basic luciferase expression vector (Promega). The 10-bp deletion variant was made via site-directed mutagenesis of the 157-bp fragment according to the manufacturer’s protocol (Q5 Site-Directed Mutagenesis Kit, New England BioLabs). After 18 h of digestion and linearization of the pGL3-Basic vector using a 10-fold excess of XhoI and HindIII (New England BioLabs), the cloned sequences were inserted into the vector via Gibson assembly (52) (New England BioLabs master mix). All five of the above constructs were then used to transform 5-alpha Competent *Escherichia coli* (New England BioLabs) per manufacturer’s protocol. Resultant *E. coli* colonies for all constructs were screened for accurate sequences, and selected colonies were grown in Luria–Bertani broth (Gibco) with 100 µg/mL Ampicillin (Sigma). Plasmid DNA stocks for the luciferase assay were then isolated from those preparations with the ZymoPURE II Plasmid Midiprep Kit (Zymo Research) or the NucleoBond Xtra Midi EF kit (Macherey-Nagel GmbH & Co.).

The complete 3′ UTR of *TAPBP* (2,066 bp) was amplified from genomic DNA, and nucleotides were added via PCR for insertion between XbaI (5′) and BamHI (3′) restriction sites for Gibson assembly with the pGL3-Control luciferase expression vector (Promega). Digestion (with XbaI and BamHI; New England BioLabs), assembly, and transformation of competent *E. coli* were performed as described above.

The 3′ UTR construct was then subjected to two rounds of site-directed mutagenesis, according to the manufacturer’s protocol (Q5 Site-Directed Mutagenesis Kit, New England BioLabs). Accurate colonies for all site-directed mutagenesis were selected and grown as described above. The initial clone included *rs59097151* and another SNP, *rs73410010*, which are in complete LD [*r*^2^ = 1, D′ = 1 (53)]. They both have G at the variable site, so both were mutated to their A variants. The products were then cloned with added poly-A sites and inserted into a pGL3 backbone containing the SV40 promoter (Fig. 4*A*). The 5′ and 3′ end restriction-site additions were made as above prior to Gibson assembly. The resultant constructs were then used to transform 5-alpha–competent *E. coli*, and accurate clones were selected and grown for DNA plasmid stocks along with the pGL3–promoter control in the same fashion as the 5′ UTR SNP constructs. Statistical significance in all luciferase assays was determined with unpaired, two-sided Student’s *t* tests.

### Luciferase Assay.

The 293T cells plated at a density of 50,000 per well, HeLa cells plated at a density of 10,000 per well, or MCF7 cells plated at a density of 250,000 per well in 24-well plates were transfected by using the HilyMax (Dojindo) reagent with the control pRL–SV40 *Renilla* Reporter Vector (Promega) for normalization and the constructs of interest described above. For 293T experiments, 250 ng of luciferase reporter plasmid and 5 ng of Renilla reporter vector were used per well. HeLa cells were transfected with 750 ng of luciferase reporter plasmid and 7.5 ng of Renilla reporter vector per well. MCF7 cells were transfected with 1 μg of luciferase reporter plasmid and 50 ng of Renilla reporter vector per well. Cells were lysed and assayed for luciferase activity with the Dual-Luciferase Reporter Assay System (Promega), according to the manufacturer’s instructions, 48 h posttransfection. Firefly luciferase activity from the experimental plasmids was normalized to the Renilla luciferase activity from the pRL-SV40 vector to account for potential differences in transfection efficiency. Resultant luciferase values were then normalized to the expression of the empty vector, pGL3-Basic. For the luciferase assays examining miRNA activity, the two 3′ UTR constructs containing only the *rs59097151* SNP and control constructs were transfected into 293T cells, as described above. For inducing additional, exogenous miRNA activity, 20 pmol per well of the miRIDIAN miR Mimic Negative Control #1 or miR-4486 mimic (Dharmacon) were cotransfected into cells. For inhibiting endogenous miRNA activity, 20 pmol per well of miRIDIAN miR Hairpin Inhibitor Negative Control #1 or the miR-4486 inhibitor (Dharmacon) were costransfected into cells.

### EMSA.

Nuclear extracts prepared from 293T, HeLa, and MCF7 cell lines via the CellLyticNuCLEAR extraction kit (Sigma-Aldrich) were stored at −80 °C until use. Five double-stranded DNA oligonucleotide probes were labeled by using the Klenow fragment of DNA polymerase I (Invitrogen) with α-[32P]deoxycytidine triphosphate (3,000 Ci/mmol; PerkinElmer Life and Analytical Sciences) and purified with mini Quick Spin Oligo Columns (Roche): the control AP-2α, the control Sp1, the *G* variant of *rs111686073*, the *C* variant of *rs111686073*, and the mutant of *rs111686073* abrogating the AP-2α binding site. (The sequences for each oligomer are listed in the *SI Appendix*, Table S7). The ^32^P-labeled oligonucleotide probe (10,000 counts per minute) was incubated for 20 min at room temperature in a total of 20 μL with the following prior to loading on a 5% polyacrylamide gel: 10 µg of nuclear extract and 1 µg of poly(2′-deoxyinosinic-2′-deoxycytidylic acid) (Sigma-Aldrich) with control IgG, Sp1, or AP-2α antibodies (Santa Cruz Biotechnology), as described (54). Electrophoresis was performed in 0.5x Tris–borate–ethylenediaminetetraacetic acid for 2 h and 30 min at 120 V. The gel was visualized with autoradiography.

### The 3′ RACE for *TAPBP* mRNA.

Samples preserved in QIAzol were added to a Phase Lock Gel Heavy spin column (5 Prime) and incubated for 5 min with GlycoBlue coprecipitant (Life Technologies) as per the manufacturer’s instructions. Chloroform was added at a 1:5 ratio and mixed in by shaking for at least 15 s prior to centrifugation at 12,000 × *g* for 10 min at 4 °C. The resultant aqueous layer was transferred to a new tube with 2 volumes of isopropanol and incubated at room temperature for 10 min to precipitate the RNA. After centrifugation with the same settings, the pellet was washed with 80% ethanol and centrifuged again prior to resuspension in molecular biology-grade distilled water.

RNA from PBMCs of three RDP donors was amplified with the 3′ RACE System (Invitrogen) for the first and nested PCRs (primer sequences provided in the *SI Appendix*, Table S7) per the manufacturer’s protocol and with Platinum Taq DNA Polymerase (Invitrogen). Amplicons were electrophoresed on 2% agarose gel, and DNA was isolated and purified from the two main bands of the nested PCR with the QIAquick Gel Extraction Kit (Qiagen). The purified DNA was sequenced with 3′ UTR-specific primers and the Abridged Universal Amplification Primer (Invitrogen).

### Quantification of miRNA Species.

RNA was isolated via QIAzol extraction as described above from 293T cells, HeLa cells, six B-cell lines homozygous for *rs59097151A* or *rs59097151G*, two B-cell lines heterozygous for *rs59097151*, and fresh PBMC samples from each of three RDP donors. The expression of miRNA species was determined via TaqMan miR Assays for miR-4486 (465336_mat), miR-423 (000576), and RNU48 (001006) with the TaqMan miR Reverse Transcription Kit and the TaqMan Fast Advanced Master Mix (Applied Biosystems). hsa-miR-423 and RNU48, a small nuclear RNA, served as controls that were stably expressed across the cell types examined (*SI Appendix*, Fig. S4*A*). By using the 2^-ΔΔCt^ method, the qPCR results were analyzed for miR-4486 expression with RNU48 as a reference for expression and miR-423 as the endogenous control.

### Tapasin-Dependence Value Measurement and Calculation.

Lentiviral vectors containing FLAG-tagged *HLA* alleles were generated and used to transfect parental tapasin-deficient 721.220 and tapasin-reconstituted 721.220 cells as described (11). Briefly, lentiviral constructs were either ordered from LifeSct or generated via site-directed mutagenesis per the manufacturer’s protocol (Q5 Site-Directed Mutagenesis Kit, New England BioLabs). These plasmids were then transfected into 293T cells along with ps-PAX2, encoding structural HIV proteins, and pHEF-VSVG envelope with Lipofectamine 2000 per the manufacturer’s instructions (Invitrogen). After 3 d, the supernatant was collected from each culture, filtered, and frozen down as virus stocks. Parental and tapasin+ 721.220 cultures were spinoculated with virus containing each of the HLA alleles for 2 h at 500 × *g*, 37 °C. Cells were cultured for 2 d at 37 °C before selection with 0.25 µg/mL puromycin for 6 d. Flow-cytometric analysis was then run once every other day on the cells and repeated at least three times. Cells were stained with anti-FLAG antibody in allophycocyanin (BioLegend) and run on a MACSQuant Analyzer 16-flow cytometer (Miltenyi Biotech). Data were analyzed with FlowJo 10, and the tapasin dependence value was calculated for each allotype as described (11). In brief, the FLAG and zsGreen median fluorescence intensities (MFIs) of the control transfections were first subtracted from the MFI for each of the cell lines of the HLA allotypes in both the 721.220 parental and tapasin+ groups to subtract background. Next, the adjusted MFIs were normalized to the average MFI of each day in that channel. The following calculation was performed for each specific allotype: (normalized HLA MFI of tapasin+ 721.220/normalized zsGreen MFI of tapasin+ 721.220)/(normalized HLA MFI of parental 721.220/normalized zsGreen MFI of parental 721.220) = tapasin-dependence value.

### Statistical Analyses.

We analyzed the association of *TAPBP* mRNA expression as determined by SNP genotypes and tapasin dependence of HLA allotypes with three different outcomes related to malaria immunity:

1. Parasite prevalence: Having at least one symptomatic or asymptomatic parasitemic (by microscopy) visit per quarter.
2. Annual malaria incidence rate: Number of symptomatic malaria episodes per person-year.
3. Parasite density: Log parasite density measured during parasitemic visits (in analyses, stratified by two visit types: routine quarterly visits and symptomatic malaria visits).

*TAPBP* mRNA expression level was estimated three ways in each individual: *rs111686073* as a dichotomous variable (*CG/GG* high expressors vs. *CC* low expressors), *rs59097151* as a dichotomous variable (*AA* high expressors vs. *AG/GG* low expressors), and expression as a continuous variable, as determined by the combination of the SNP genotypes into three groups based on Fig. 1*D* (*CC*/*GG* and *CC*/*AG* in group 1; *CG*/*AG* and *CC*/*AA* in group 2; and *CG*/*AA* and *GG*/*AA* in group 3). Tapasin dependence was estimated over the three HLA loci combined (HLA-A, HLA-B, and HLA-C), or per each individual locus, and used as a continuous or a binary variable. The distribution of tapasin dependence is heterogeneous, so arbitrary cutoffs were used to delineate tapasin-dependent from -independent genotypes, ensuring good representation in each group (*SI Appendix*, Fig. S5).

The impact of tapasin dependence and *TAPBP* mRNA expression, stratified or not by tapasin-dependence level, on the various outcomes were estimated through multilevel models with random effects at the individual and household levels. We used logistic regression for parasite prevalence, Poisson models for malaria incidence, and linear models for log parasite density. All models were adjusted for site, household log EIR, and sex. Controlling for ethnicity (Bantu/non-Bantu) based on the language of consent form did not improve model fit, as compared to controlling for site due to collinearity. Models included a continuous linear term for age, a binary indicator of age group (child vs. adult), and an interaction between the two to allow for effects to vary between age groups because the study did not enroll participants between the ages of 11 and 17. In most multivariable models, adults had a lower burden and households with higher log EIR had a higher burden of both incident malaria and parasite prevalence (*SI Appendix*, Table S1). Those in Jinja tended to have a lower risk of incident malaria (but not parasite prevalence) than those in Tororo (reference site), even after controlling for all covariates. In parasite prevalence models, the interaction term between adult and age was also generally statistically significant.

In summary, the models followed this general form (an example model for parasite prevalence shown):

$$\text{logit}\left(\text{Parasite}\;{\text{prevalence}}_{\mathrm{ijk}}\right)=\text{TAPBP}\;\text{mRNA}\;\text{expression/dependence}\;{\text{genotype}}_{ij}+{\text{Site}}_{j}+\text{Log}\left(\mathrm{aEI}R_{j}\right)+{\text{Sex}}_{ij}+{\text{Age}}_{\mathrm{ijk}}+{\text{Child}}_{ij}+\text{Age}*{\text{Child}}_{\mathrm{ijk}}+u_{i}+y_{j}\text{,}$$

where *j* indicates households, *i* indicates individuals, and *k* indicates specific visits. For example, Age*_ijk_* denotes the age of child *i* from household *j* during visit *k*. As *aEIR_j_* represents the average annual EIR recorded for household *j* (time-invariant), we assume relatively stable transmission intensity over the course of study follow-up.

The CIs determined from these models were used in Figs. 6–8. To further validate the *P* values, we generated empirical *P* values using Monte Carlo permutation tests. We permuted exposure variables 10,000 times and calculated the two-sided *P* value as the number of permutations that yielded coefficients greater than or equal to the absolute value of the observed coefficient divided by the number of permutations (excluding those that did not converge; <1%) using the Stata ritest package v1.1.4 (55). If the calculated *P* value was equal to zero, we made the conservative assumption that it was one divided by the number of permutations. We controlled for multiple testing separately for each outcome using the FDR approach to generate q-values, indicated in Figs. 6–8, with the Stata qqvalue package (56). All analyses were done in Stata 15.1 (StataCorp, 2017).

## Supplementary Material

Supplementary File

## Data Availability

Anonymized data and code used in the association analyses involving the parallel malaria cohort studies have been deposited in GitHub (https://github.com/feeneylab/TAPBPmalaria) (57) and are publicly accessible. All other raw data displayed in the figures and supplementary information are available upon reasonable request.

## References

[1] S. Hulpke, R. Tampé, The MHC I loading complex: A multitasking machinery in adaptive immunity. Trends Biochem. Sci. 38, 412–420 (2013). 23849087 10.1016/j.tibs.2013.06.003

[2] M. Chen, M. Bouvier, Analysis of interactions in a tapasin/class I complex provides a mechanism for peptide selection. EMBO J. 26, 1681–1690 (2007). 17332746 10.1038/sj.emboj.7601624 PMC1829385

[3] O. Fisette, G. F. Schröder, L. V. Schäfer, Atomistic structure and dynamics of the human MHC-I peptide-loading complex. Proc. Natl. Acad. Sci. U.S.A. 117, 20597–20606 (2020). 32788370 10.1073/pnas.2004445117 PMC7456110

[4] I. Hafstrand , Successive crystal structure snapshots suggest the basis for MHC class I peptide loading and editing by tapasin. Proc. Natl. Acad. Sci. U.S.A. 116, 5055–5060 (2019). 30808808 10.1073/pnas.1807656116 PMC6421438

[5] M. Howarth, A. Williams, A. B. Tolstrup, T. Elliott, Tapasin enhances MHC class I peptide presentation according to peptide half-life. Proc. Natl. Acad. Sci. U.S.A. 101, 11737–11742 (2004). 15286279 10.1073/pnas.0306294101 PMC511045

[6] P. A. Wearsch, P. Cresswell, Selective loading of high-affinity peptides onto major histocompatibility complex class I molecules by the tapasin-ERp57 heterodimer. Nat. Immunol. 8, 873–881 (2007). 17603487 10.1038/ni1485

[7] T. H. Hansen, M. Bouvier, MHC class I antigen presentation: Learning from viral evasion strategies. Nat. Rev. Immunol. 9, 503–513 (2009). 19498380 10.1038/nri2575

[8] I. B. Harvey, X. Wang, D. H. Fremont, Molluscum contagiosum virus MC80 sabotages MHC-I antigen presentation by targeting tapasin for ER-associated degradation. PLoS Pathog. 15, e1007711 (2019). 31034515 10.1371/journal.ppat.1007711 PMC6508746

[9] Y. Shionoya , Loss of tapasin in human lung and colon cancer cells and escape from tumor-associated antigen-specific CTL recognition. OncoImmunology 6, e1274476 (2017). 28344889 10.1080/2162402X.2016.1274476 PMC5353923

[10] C. Thuring, L. Geironson, K. Paulsson, Tapasin and human leukocyte antigen class I dysregulation correlates with survival in glioblastoma multiforme. Anticancer. Agents Med. Chem. 14, 1101–1109 (2014). 25175688 10.2174/1871520614666140825110402

[11] A. A. Bashirova , HLA tapasin independence: Broader peptide repertoire and HIV control. Proc Natl Acad Sci U S A 117, 28232–28238 (2020). 33097667 10.1073/pnas.2013554117 PMC7668082

[12] R. Greenwood, Y. Shimizu, G. S. Sekhon, R. DeMars, Novel allele-specific, post-translational reduction in HLA class I surface expression in a mutant human B cell line. J. Immunol. 153, 5525–5536 (1994). 7989754

[13] C. A. Peh , HLA-B27-restricted antigen presentation in the absence of tapasin reveals polymorphism in mechanisms of HLA class I peptide loading. Immunity 8, 531–542 (1998). 9620674 10.1016/s1074-7613(00)80558-0

[14] S. M. Rizvi , Distinct assembly profiles of HLA-B molecules. J. Immunol. 192, 4967–4976 (2014). 24790147 10.4049/jimmunol.1301670 PMC4117407

[15] A. P. Williams, C. A. Peh, A. W. Purcell, J. McCluskey, T. Elliott, Optimization of the MHC class I peptide cargo is dependent on tapasin. Immunity 16, 509–520 (2002). 11970875 10.1016/s1074-7613(02)00304-7

[16] D. Zernich , Natural HLA class I polymorphism controls the pathway of antigen presentation and susceptibility to viral evasion. J. Exp. Med. 200, 13–24 (2004). 15226359 10.1084/jem.20031680 PMC2213310

[17] A. Bailey , Selector function of MHC I molecules is determined by protein plasticity. Sci. Rep. 5, 14928 (2015). 26482009 10.1038/srep14928 PMC5224517

[18] M. A. Garstka , Tapasin dependence of major histocompatibility complex class I molecules correlates with their conformational flexibility. FASEB J. 25, 3989–3998 (2011). 21836024 10.1096/fj.11-190249

[19] L. A. McPherson, R. J. Weigel, AP2alpha and AP2gamma: A comparison of binding site specificity and trans-activation of the estrogen receptor promoter and single site promoter constructs. Nucleic Acids Res. 27, 4040–4049 (1999). 10497269 10.1093/nar/27.20.4040 PMC148672

[20] W. Liu, X. Wang, Prediction of functional microRNA targets by integrative modeling of microRNA binding and target expression data. Genome Biol. 20, 18 (2019). 30670076 10.1186/s13059-019-1629-z PMC6341724

[21] N. Wong, X. Wang, miRDB: An online resource for microRNA target prediction and functional annotations. Nucleic Acids Res. 43, D146–D152 (2015). 25378301 10.1093/nar/gku1104 PMC4383922

[22] V. Agarwal, G. W. Bell, J. W. Nam, D. P. Bartel, Predicting effective microRNA target sites in mammalian mRNAs. eLife 4, e05005 (2015). 10.7554/eLife.05005 PMC4532895 26267216

[23] M. Rehmsmeier, P. Steffen, M. Hochsmann, R. Giegerich, Fast and effective prediction of microRNA/target duplexes. RNA 10, 1507–1517 (2004). 15383676 10.1261/rna.5248604 PMC1370637

[24] N. Ludwig , Distribution of miRNA expression across human tissues. Nucleic Acids Res. 44, 3865–3877 (2016). 26921406 10.1093/nar/gkw116 PMC4856985

[25] J. C. Digitale , Inhibitory KIR ligands are associated with higher *P. falciparum* parasite prevalence. J. Infect. Dis. 224, 175–183 (2021). 33165540 10.1093/infdis/jiaa698 PMC8491837

[26] A. Katureebe , Measures of malaria burden after long-lasting insecticidal net distribution and indoor residual spraying at three sites in Uganda: A prospective observational study. PLoS Med. 13, e1002167 (2016). 27824885 10.1371/journal.pmed.1002167 PMC5100985

[27] M. R. Kamya , Malaria transmission, infection, and disease at three sites with varied transmission intensity in Uganda: Implications for malaria control. Am. J. Trop. Med. Hyg. 92, 903–912 (2015). 25778501 10.4269/ajtmh.14-0312 PMC4426576

[28] E. E. Davenport , Genomic landscape of the individual host response and outcomes in sepsis: A prospective cohort study. Lancet Respir. Med. 4, 259–271 (2016). 26917434 10.1016/S2213-2600(16)00046-1 PMC4820667

[29] B. P. Fairfax , Genetics of gene expression in primary immune cells identifies cell type-specific master regulators and roles of HLA alleles. Nat. Genet. 44, 502–510 (2012). 22446964 10.1038/ng.2205 PMC3437404

[30] S. Ashraf , Synergism of tapasin and human leukocyte antigens in resolving hepatitis C virus infection. Hepatology 58, 881–889 (2013). 23532923 10.1002/hep.26415 PMC3759612

[31] J. Shao , Targeted re-sequencing identified rs3106189 at the 5′ UTR of TAPBP and rs1052918 at the 3′ UTR of TCF3 to be associated with the overall survival of colorectal cancer patients. PLoS One 8, e70307 (2013). 23940558 10.1371/journal.pone.0070307 PMC3734069

[32] A. W. Purcell , Quantitative and qualitative influences of tapasin on the class I peptide repertoire. J. Immunol. 166, 1016–1027 (2001). 11145681 10.4049/jimmunol.166.2.1016

[33] D. S. Boulanger , Absence of tapasin alters immunodominance against a lymphocytic choriomeningitis virus polytope. J. Immunol. 184, 73–83 (2010). 19949070 10.4049/jimmunol.0803489

[34] S. P. Kurup, N. S. Butler, J. T. Harty, T cell-mediated immunity to malaria. Nat. Rev. Immunol. 19, 457–471 (2019). 30940932 10.1038/s41577-019-0158-z PMC6599480

[35] C. A. Long, F. Zavala, Immune responses in Malaria. Cold Spring Harb. Perspect. Med. 7, a025577 (2017). 28389518 10.1101/cshperspect.a025577 PMC5538407

[36] D. Fernandez-Ruiz , Liver-resident memory CD8^+^ T Cells form a front-line defense against malaria liver-stage infection. Immunity 45, 889–902 (2016). 27692609 10.1016/j.immuni.2016.08.011

[37] R. A. Seder ; VRC 312 Study Team, Protection against malaria by intravenous immunization with a nonreplicating sporozoite vaccine. Science 341, 1359–1365 (2013). 23929949 10.1126/science.1241800

[38] P. Bejon , Analysis of immunity to febrile malaria in children that distinguishes immunity from lack of exposure. Infect. Immun. 77, 1917–1923 (2009). 19223480 10.1128/IAI.01358-08 PMC2681775

[39] J. C. Digitale , HLA alleles B^*^53:01 and C^*^06:02 are associated with higher risk of *P. falciparum* parasitemia in a cohort in Uganda. Front. Immunol. 12, 650028 (2021). 33815410 10.3389/fimmu.2021.650028 PMC8017319

[40] J. A. Ake ; African Cohort Study Team, Noninfectious comorbidity in the African cohort study. Clin. Infect. Dis. 69, 639–647 (2019). 30476001 10.1093/cid/ciy981 PMC6669288

[41] A. Kakuru , Dihydroartemisinin-piperaquine for the prevention of malaria in pregnancy. N. Engl. J. Med. 374, 928–939 (2016). 26962728 10.1056/NEJMoa1509150 PMC4847718

[42] I. Rodriguez-Barraquer , Quantification of anti-parasite and anti-disease immunity to malaria as a function of age and exposure. eLife 7, e35832 (2018). 30044224 10.7554/eLife.35832 PMC6103767

[43] M. Kilama , Estimating the annual entomological inoculation rate for *Plasmodium falciparum* transmitted by *Anopheles gambiae s.l.* using three sampling methods in three sites in Uganda. Malar. J. 13, 111 (2014). 24656206 10.1186/1475-2875-13-111 PMC4001112

[44] P. K. Ehrenberg , A vaccine-induced gene expression signature correlates with protection against SIV and HIV in multiple trials. Sci. Transl. Med. 11, eaaw4236 (2019). 31462510 10.1126/scitranslmed.aaw4236 PMC7383941

[45] S. Shangguan , Monocyte-derived transcriptome signature indicates antibody-dependent cellular phagocytosis as a potential mechanism of vaccine-induced protection against HIV-1. eLife 10, e69577 (2021). 34533134 10.7554/eLife.69577 PMC8514236

[46] S. Chen, Y. Zhou, Y. Chen, J. Gu, fastp: An ultra-fast all-in-one FASTQ preprocessor. Bioinformatics 34, i884–i890 (2018). 30423086 10.1093/bioinformatics/bty560 PMC6129281

[47] A. M. Bolger, M. Lohse, B. Usadel, Trimmomatic: A flexible trimmer for Illumina sequence data. Bioinformatics 30, 2114–2120 (2014). 24695404 10.1093/bioinformatics/btu170 PMC4103590

[48] D. Kim, B. Langmead, S. L. Salzberg, HISAT: A fast spliced aligner with low memory requirements. Nat. Methods 12, 357–360 (2015). 25751142 10.1038/nmeth.3317 PMC4655817

[49] S. Anders, P. T. Pyl, W. Huber, HTSeq—A Python framework to work with high-throughput sequencing data. Bioinformatics 31, 166–169 (2015). 25260700 10.1093/bioinformatics/btu638 PMC4287950

[50] M. D. Robinson, D. J. McCarthy, G. K. Smyth, edgeR: A Bioconductor package for differential expression analysis of digital gene expression data. Bioinformatics 26, 139–140 (2010). 19910308 10.1093/bioinformatics/btp616 PMC2796818

[51] P. Danecek , Twelve years of SAMtools and BCFtools. Gigascience 10, giab008 (2021). 33590861 10.1093/gigascience/giab008 PMC7931819

[52] D. G. Gibson , Enzymatic assembly of DNA molecules up to several hundred kilobases. Nat. Methods 6, 343–345 (2009). 19363495 10.1038/nmeth.1318

[53] A. Auton ; 1000 Genomes Project Consortium, A global reference for human genetic variation. Nature 526, 68–74 (2015). 26432245 10.1038/nature15393 PMC4750478

[54] H. Li, P. W. Wright, M. McCullen, S. K. Anderson, Characterization of KIR intermediate promoters reveals four promoter types associated with distinct expression patterns of KIR subtypes. Genes Immun. 17, 66–74 (2016). 26656451 10.1038/gene.2015.56 PMC4724278

[55] S. Heß, Randomization inference with Stata: A guide and software. Stata J. 17, 630–651 (2017).

[56] R. B. Newson, Frequentist Q-values for multiple-test procedures. Stata J. 10, 568–584 (2010).

[57] V. Walker-Sperling ., Genetic variation that determines TAPBP expression levels associates with the course of malaria in an HLA allotype-dependent manner. TAPBPmalaria. Github. https://github.com/feeneylab/TAPBPmalaria. Deposited 17 February 2022. 10.1073/pnas.2205498119 PMC9303992 35858344

