# Supplementary material for: Genetic variation that determines TAPBP expression levels associates with the course of malaria in an HLA allotype-dependent manner
Source: Proc Natl Acad Sci U S A. 2022 Jul 13;119(29):e2205498119. doi: 10.1073/pnas.2205498119 (PMC9303992; doi:10.1073/pnas.2205498119)
Supplement: Supplementary File [file pnas.2205498119.sapp.pdf]

PNAS

**Supplementary Information for**
# Genetic variation that determines *TAPBP* expression levels associates with the course of malaria in an HLA allotype-dependent manner.

Victoria Walker-Sperling[1†‡], Jean C. Digitale[2,3‡], Mathias Viard[1,4], Maureen P. Martin[1,4], Arman Bashirova[1,4], Yuko Yuki[1,4], Veron Ramsuran[5], Smita Kulkarni[6], Vivek Naranbhai[5,7-10], Hongchuan Li[4,11], Stephen K. Anderson[4,11], Lauren Yum[12,13], Robert Clifford[12,13], Hannah Kibuuka[14], Julie Ake[12], Rasmi Thomas[12], Sarah Rowland-Jones[15], John Rek[16], Emmanuel Arinaitwe[16], Moses Kamya[16,17], Isabel Rodriguez-Barraquer[2], Margaret E. Feeney[2,18], Mary Carrington[1,4,19*]

Corresponding author: Mary Carrington.
**Email:** carringm@mail.nih.gov

**This PDF file includes:**

Figures S1 to S6
Tables S1 to S6
SI References

| Allele Frequencies: | | Rare Allele | Common Allele |
|---|---|---|---|
| rs111686073 | 5' UTR | 16.4% G (h) | 83.6% C (l) * |
| rs59097151 | 3' UTR | 18.1% G (l) | 81.9% A (h) * |

| Haplotypes (D' = 1, $r^2 < 0.05$): rs111686073/rs59097151 | Haplotype Expression Level | Haplotype Frequency |
|---|---|---|
| C/G | Low/Low | 18.1% |
| C/A * | Low/High | 65.5% |
| G/A | High/High | 16.4% |

*Alleles & haplotypes fixed in white populations.

**Fig. S1.** Allele and haplotype frequencies of *rs111686073* and *rs59097151* in the Ugandan malaria cohorts. The general location, allele frequency, and relative expression level of each SNP is indicated in the upper table. The haplotypes (indicated as *rs111686073* variant/*rs59097151* variant) are shown with the relative expression level combinations (h=high and l=low) and haplotype frequencies. *Allele/haplotype fixed in White populations.

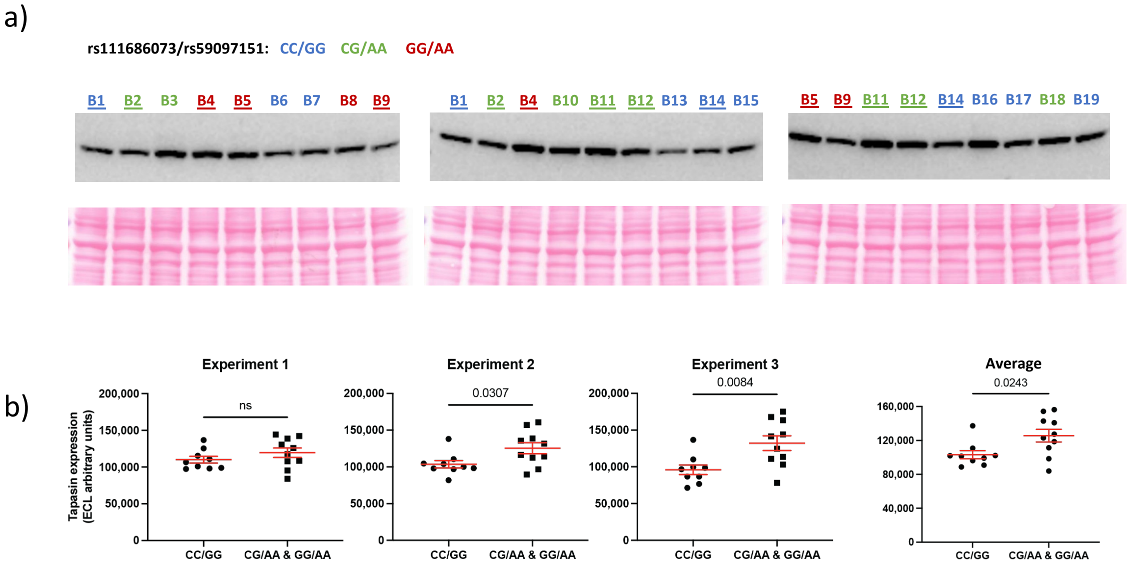


**Fig. S2.** Western blot analysis of tapasin protein expression in 19 B lymphoblastoid cell lines (BLCLs). A) Representative experiment showing ECL images (top) and Ponceau S staining (bottom) confirming equal loading. Total cell lysates from 19 BLCLs (B1 – B19) were loaded on three gels. Each lane is labeled with a corresponding BLCL, and the labels are color-coded according to tapasin genotypes, rs111686073/rs59097151, as shown above. Underlined labels indicate BLCL cell lysates that were loaded on two gels for cross-reference. B) Tapasin quantification in three separate Western blot experiments. Data for each experiment was normalized to average signal value. The graph on the right shows data averaged for the three experiments. The red bars indicate the mean ± the standard error (SEM). P-values for unpaired t-tests are shown for each graph. Significantly higher tapasin protein expression is observed in BLCL lysates from subjects with at least one *rs111686073G* (i.e. *CG* or *GG*) variant along with the *rs59097151AA* genotype.

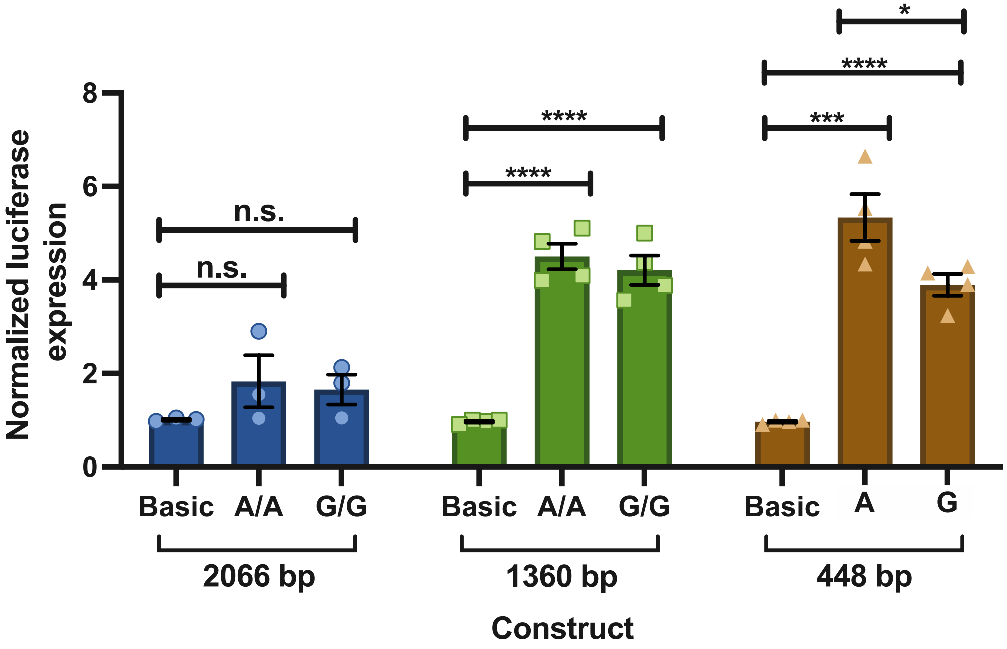


**Fig. S3.** Full 3'UTR in constructs inhibit luciferase expression compared to progressively shortened constructs. pGL3-based constructs with either the full (2066 bp with original polyadenylation site) or shortened 3'UTR sequences of *TAPBP* were constructed using Gibson assembly and site-directed mutagenesis (1360 bp and 448 bp with the AATAAA poly-A tail signal added at the ends; detailed description of constructs are shown in Figure 4A). The constructs were transfected into 293T cells, and after two days, cells were lysed, and luciferase activity was read and normalized to the expression of Renilla and the empty pGL3-basic vector (Promega). Luciferase expression of the full 3'UTR construct was not significantly different than pGL3-basic, the negative control. Each symbol represents a single independent experiment with (three for the 2066 bp construct and four for the other two), and the bars indicate the mean ± the standard error (SEM). Significance calculated with unpaired, two-sided Student's t-tests. *$p<0.05$, ***$p < 0.001$, ****$p < 0.0001$.

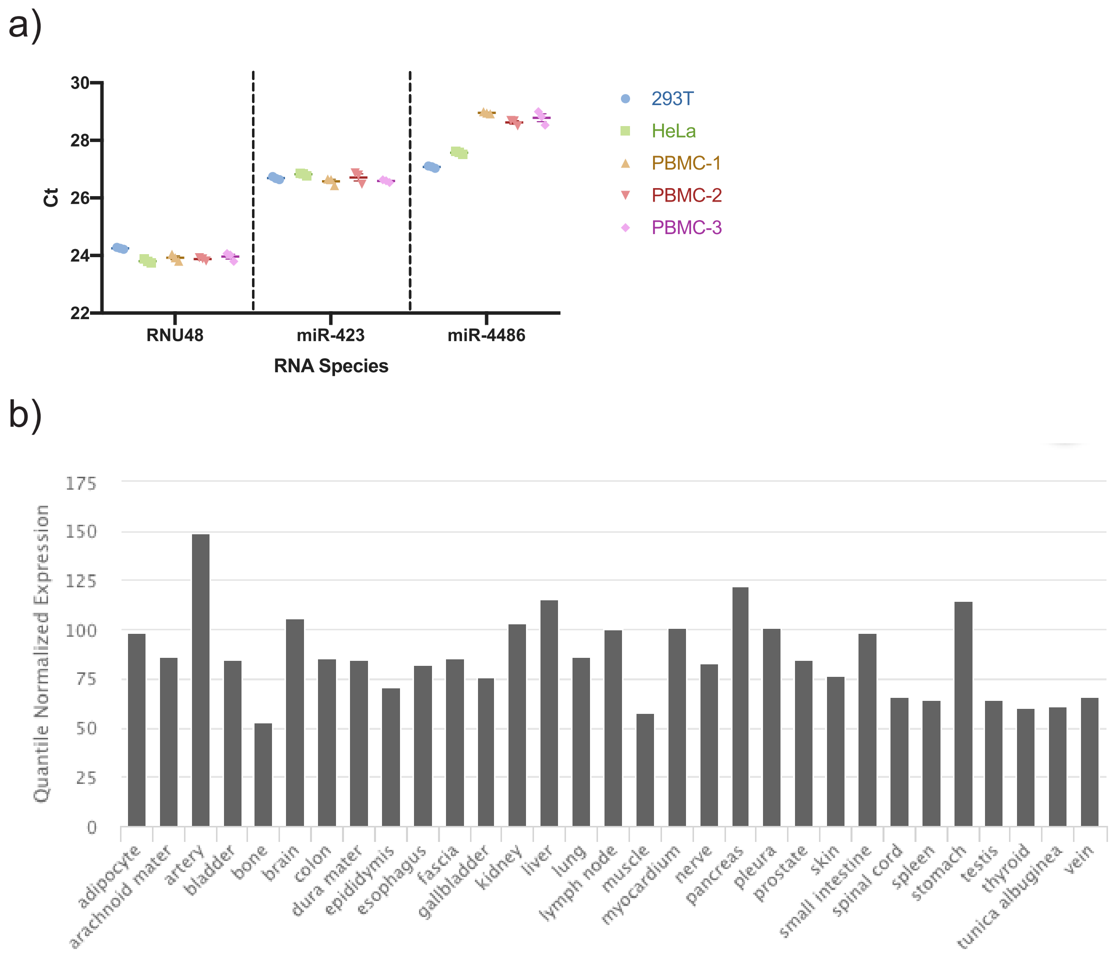


**Fig. S4.** Expression of miR-4486 in various cell types. A) Expression of mature miR-4486 and reference small RNA species (RNU48 & miR-423) as measured using TaqMan MicroRNA qPCR assays (mean ± SEM). These results were taken into account for Figure 5B where expression was normalized to miR-423 to correct for differences in RNA sampling and sample variation, followed by normalization to the expression of RNU48 (used as reference), a well-expressed small nuclear RNA, using the $2^{-\Delta\Delta Ct}$ method of quantification. B) Expression of miR-4486 across different tissue types according to the online miRNA expression database Tissue Atlas(1).

a)

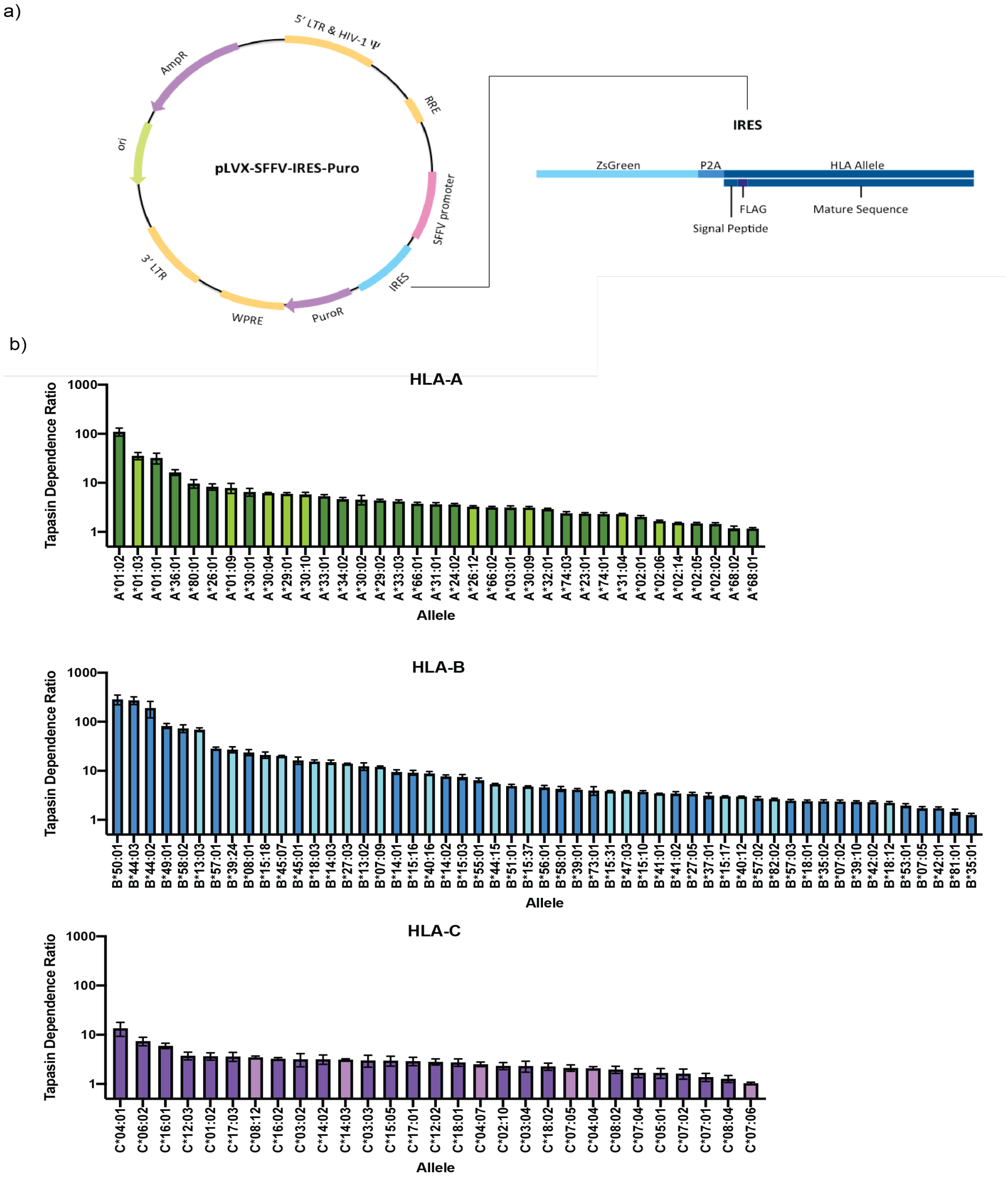


**Figure S5.** Tapasin dependence values for allotypes present in the malaria cohort. Each *HLA* allele was inserted into a pLVX-SFFV-IRES-Puro vector(2) with a FLAG tag added between the signal peptide and mature protein coding sequence. The constructs were transfected into parental 721.220 (tapasin deficient) cells and 721.220 cells reconsitituted with tapasin. The MFI of the FLAG-tagged allotypes was measured by flow cytometry in at least triplicate, and the tapasin dependence ratio was calculated as described previously(2). A) Diagram of the construct and arrangement of the protein coding sequences with the P2A cleavage site between ZsGreen and the HLA notated. B) Tapasin dependence ratios for HLA-A, -B, and -C allotypes (non-log transformed; mean ± SEM). The darker bars indicate values calculated previously (n=83)(2), and the lighter bars indicate allotypes that were determined for this study (n=34).

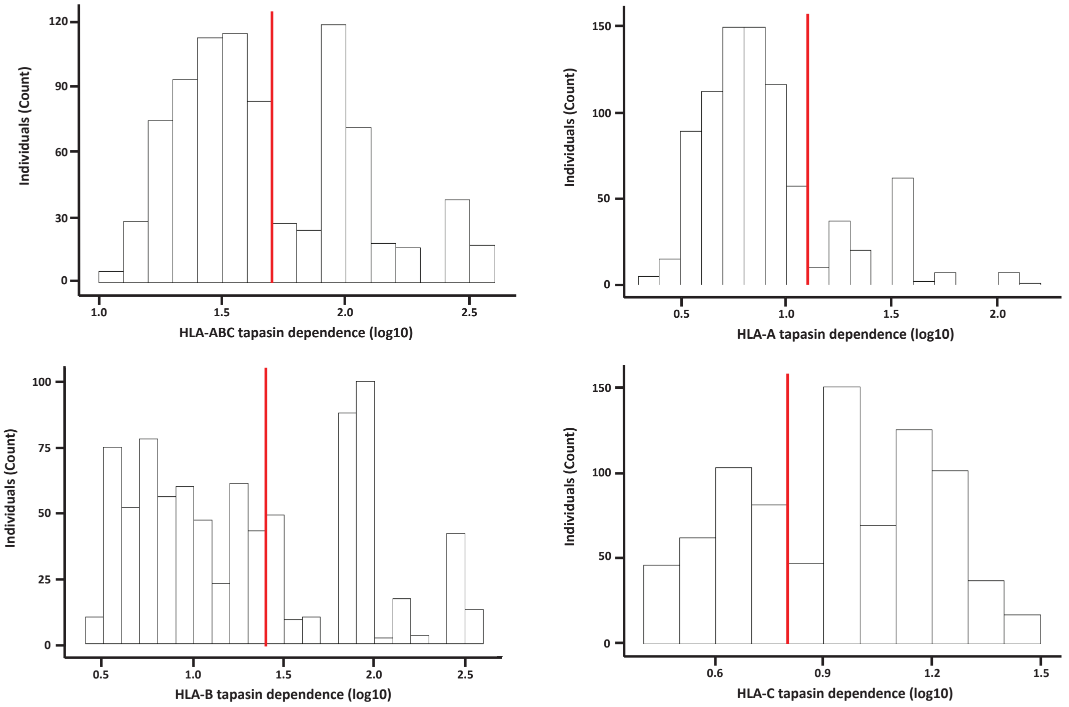


**Figure S6.** Empirical HLA tapasin dependence value cut-offs.  The full malaria cohort was examined for the tapasin dependence of their HLA allotypes, and cut-offs, shown with a red line, between high and low dependence were determined empirically

**Table S1.** The relationship of covariates in the multivariable models examining *TAPBP* mRNA i-expression to malaria outcome measures. All model coefficients are included for the results for which false discovery rate q-value < 0.05. The location of the analyses is indicated in the first column, “Table” (ex. the continuous *TAPBP* mRNA i-expression analysis is found in Table S3).

| Table | HLA locus | Exposure | Among | Exposure | Exposure (p) | Age (OR) | Age (p) | Adult (OR) | Adult (p) | Interaction Adult x Age (OR) | Interaction Adult x Age (p) | Male (OR) | Male (p) | log EIR (OR) | log EIR (p) | Jinja (OR) | Jinja (p) | Kanungu (OR) | Kanungu (p) | Tororo (OR) | Tororo (p) | N (participants) |
|---|---|---|---|---|---|---|---|---|---|---|---|---|---|---|---|---|---|---|---|---|---|---|
| **Incident Malaria** | | | | | | | | | | | | | | | | | | | | | | |
| S5 | HLA-ABC | Continous tapasin i-expression | Dependent | 0.541 | 0.000 | 0.982 | 0.112 | 1.432 | 0.452 | 1.061 | 0.005 | 1.086 | 0.464 | 1.419 | 0.001 | 0.340 | 0.010 | 1.215 | 0.492 | 1 | NA | 328 |
| S5 | HLA-B | Continous tapasin i-expression | Dependent | 0.530 | **0.000** | 0.991 | 0.429 | 2.519 | **0.039** | 1.033 | 0.118 | 1.094 | 0.406 | 1.435 | **0.000** | 0.421 | **0.031** | 1.471 | 0.162 | 1 | NA | 332 |
| S5 | HLA-C | Continous tapasin i-expression | Dependent | 0.714 | **0.004** | 0.999 | 0.882 | 3.986 | **0.000** | 1.006 | 0.700 | 1.066 | 0.474 | 1.321 | **0.002** | 0.408 | **0.008** | 1.203 | 0.437 | 1 | NA | 543 |
| S5 | HLA-ABC | Tapasin i-expression level based on rs59097151 genotype | Dependent | 0.557 | **0.000** | 0.980 | 0.077 | 1.319 | 0.563 | 1.063 | **0.004** | 1.065 | 0.575 | 1.431 | **0.000** | 0.354 | **0.011** | 1.356 | 0.269 | 1 | NA | 328 |
| S5 | HLA-B | Tapasin i-expression level based on rs59097151 genotype | Dependent | 0.610 | **0.000** | 0.990 | 0.375 | 2.467 | **0.045** | 1.031 | 0.136 | 1.067 | 0.549 | 1.439 | **0.000** | 0.424 | **0.031** | 1.557 | 0.107 | 1 | NA | 332 |
| S5 | HLA-B | Tapasin i-expression level based on rs59097151 genotype | Dependent | 0.749 | **0.003** | 0.998 | 0.818 | 3.921 | **0.000** | 1.006 | 0.721 | 1.060 | 0.518 | 1.322 | **0.002** | 0.419 | **0.010** | 1.255 | 0.336 | 1 | NA | 543 |
| S5 | HLA-B | Tapasin i-expression level based on rs111686073 genotype | Dependent | 0.717 | **0.024** | 0.993 | 0.550 | 2.882 | **0.018** | 1.027 | 0.201 | 1.068 | 0.549 | 1.432 | **0.000** | 0.431 | **0.040** | 1.392 | 0.246 | 1 | NA | 332 |
| S6 | HLA-ABC | Low i-expression & Dependence vs. Independence | All | 1.428 | **0.002** | 1.004 | 0.598 | 5.503 | **0.000** | 0.991 | 0.486 | 1.043 | 0.537 | 1.379 | **0.000** | 0.448 | **0.005** | 1.296 | 0.195 | 1 | NA | 833 |
| S6 | HLA-B | Low i-expression & Dependence vs. Independence | All | 1.342 | **0.009** | 1.004 | 0.582 | 5.525 | **0.000** | 0.991 | 0.506 | 1.044 | 0.526 | 1.388 | **0.000** | 0.455 | **0.006** | 1.315 | 0.173 | 1 | NA | 833 |
| S6 | HLA-C | Low i-expression & Dependence vs. Independence | All | 1.292 | **0.010** | 1.004 | 0.589 | 5.621 | **0.000** | 0.991 | 0.481 | 1.039 | 0.581 | 1.378 | **0.000** | 0.449 | **0.006** | 1.273 | 0.228 | 1 | NA | 833 |
| **Parasite Prevalence** | | | | | | | | | | | | | | | | | | | | | | |
| S3 | NA | Continuous TAPBP mRNA i-expression | All | 0.722 | **0.006** | 0.996 | 0.616 | 3.105 | **0.000** | 1.104 | **0.000** | 1.065 | 0.476 | 1.773 | **0.000** | 0.629 | 0.197 | 1.132 | 0.628 | 1 | NA | 835 |
| S3 | NA | rs59097151 genotype | All | 0.781 | **0.012** | 0.996 | 0.576 | 3.072 | **0.000** | 1.103 | **0.000** | 1.064 | 0.485 | 1.776 | **0.000** | 0.642 | 0.218 | 1.182 | 0.512 | 1 | NA | 835 |
| S4 | HLA-C | Continuous tapasin dependence | All | 1.547 | **0.017** | 0.996 | 0.602 | 3.071 | **0.000** | 1.103 | **0.000** | 1.055 | 0.542 | 1.809 | **0.000** | 0.681 | 0.293 | 1.203 | 0.477 | 1 | NA | 835 |
| S5 | HLA-ABC | Continous tapasin i-expression | Dependent | 0.492 | **0.000** | 0.971 | 0.017 | 0.671 | 0.428 | 1.175 | **0.000** | 1.098 | 0.487 | 1.636 | **0.000** | 0.331 | **0.032** | 0.866 | 0.691 | 1 | NA | 328 |
| S5 | HLA-ABC | Tapasin i-expression level based on rs59097151 genotype | Dependent | 0.520 | **0.000** | 0.969 | 0.010 | 0.614 | 0.335 | 1.176 | **0.000** | 1.073 | 0.600 | 1.650 | **0.000** | 0.348 | **0.038** | 0.975 | 0.944 | 1 | NA | 328 |
| S5 | HLA-A | Continous tapasin i-expression | Dependent | 0.504 | **0.019** | 1.005 | 0.811 | 1.693 | 0.597 | 1.218 | **0.000** | 1.154 | 0.501 | 2.269 | **0.000** | 0.553 | 0.431 | 0.952 | 0.924 | 1 | NA | 143 |
| S5 | HLA-A | Continous tapasin i-expression | Independent | 0.741 | **0.020** | 0.994 | 0.442 | 3.368 | **0.000** | 1.077 | **0.000** | 1.096 | 0.356 | 1.679 | **0.000** | 0.571 | 0.134 | 1.038 | 0.889 | 1 | NA | 692 |
| S5 | HLA-B | Continous tapasin i-expression | Dependent | 0.470 | **0.000** | 0.983 | 0.136 | 1.269 | 0.609 | 1.146 | **0.000** | 1.080 | 0.555 | 1.742 | **0.000** | 0.552 | 0.231 | 1.154 | 0.689 | 1 | NA | 332 |
| S5 | HLA-B | Tapasin i-expression level based on rs59097151 genotype | Dependent | 0.564 | **0.000** | 0.982 | 0.113 | 1.242 | 0.644 | 1.144 | **0.000** | 1.051 | 0.702 | 1.744 | **0.000** | 0.553 | 0.232 | 1.221 | 0.577 | 1 | NA | 332 |
| S5 | HLA-B | Tapasin i-expression level based on rs111686073 genotype | Dependent | 0.649 | **0.011** | 0.985 | 0.197 | 1.455 | 0.425 | 1.140 | **0.000** | 1.053 | 0.698 | 1.732 | **0.000** | 0.546 | 0.229 | 1.055 | 0.882 | 1 | NA | 332 |
| S5 | HLA-C | Continous tapasin i-expression | Dependent | 0.579 | **0.000** | 0.988 | 0.190 | 1.882 | 0.114 | 1.120 | **0.000** | 1.197 | 0.121 | 1.621 | **0.000** | 0.452 | 0.060 | 0.923 | 0.793 | 1 | NA | 544 |
| S5 | HLA-C | Tapasin i-expression level based on rs59097151 genotype | Dependent | 0.639 | **0.000** | 0.987 | 0.154 | 1.840 | 0.128 | 1.119 | **0.000** | 1.180 | 0.152 | 1.623 | **0.000** | 0.471 | 0.075 | 0.988 | 0.969 | 1 | NA | 544 |
| S5 | HLA-C | Tapasin i-expression level based on rs111686073 genotype | Dependent | 0.753 | **0.039** | 0.989 | 0.233 | 1.954 | 0.095 | 1.118 | **0.000** | 1.196 | 0.125 | 1.643 | **0.000** | 0.470 | 0.080 | 0.941 | 0.844 | 1 | NA | 544 |
| S6 | HLA-ABC | Low i-expression & Dependence vs. Independence | All | 1.443 | **0.013** | 0.995 | 0.531 | 2.940 | **0.001** | 1.104 | **0.000** | 1.069 | 0.450 | 1.788 | **0.000** | 0.647 | 0.229 | 1.197 | 0.486 | 1 | NA | 835 |
| S6 | HLA-B | Low i-expression & Dependence vs. Independence | All | 1.475 | **0.007** | 0.996 | 0.570 | 2.963 | **0.001** | 1.105 | **0.000** | 1.069 | 0.448 | 1.801 | **0.000** | 0.660 | 0.252 | 1.209 | 0.464 | 1 | NA | 835 |
| S6 | HLA-C | Low i-expression & Dependence vs. Independence | All | 1.571 | **0.000** | 0.995 | 0.485 | 2.891 | **0.001** | 1.106 | **0.000** | 1.063 | 0.485 | 1.790 | **0.000** | 0.654 | 0.236 | 1.178 | 0.519 | 1 | NA | 835 |

Results shown for all coefficients for which false discovery rate q-value < 0.05.

**Table S2.** Distribution of genotypes and *TAPBP* i-expression groupings. The frequency of the *rs111686973* and *rs59097151* genotypes varied somewhat by site, but the variation did not correspond to either the hierarchy of malaria incidence or entomological inoculation rate.

| | **Total N=835 N (%)** | **Site** | | | **Age** | |
|---|---|---|---|---|---|---|
| | | **Jinja** | **Kanungu** | **Tororo** | **Child** | **Adult** |
| **rs59097151 high expressors** | 572 (68.5) | 184 (68.9) | 235 (73.7) | 153 (61.5) | 425 (68.4) | 147 (68.7) |
| **rs111686073 high expressors** | 229 (27.4) | 86 (32.2) | 51 (16.0) | 92 (37.0) | 172 (27.7) | 57 (26.6) |
| **rs59097151 and rs111686073 combined** | | | | | | |
| Low expressors | 230 (27.5) | 71 (26.6) | 80 (25.1) | 79 (31.7) | 167 (26.9) | 63 (29.4) |
| Average expressors | 409 (49.0) | 122 (45.7) | 192 (60.2) | 95 (38.2) | 311 (50.1) | 98 (45.8) |
| High expressors | 196 (23.5) | 74 (27.7) | 47 (14.7) | 75 (30.1) | 143 (23.0) | 53 (24.8) |

**Table S3.** Higher *TAPBP* mRNA i-expression associates with protection against malaria incidence and parasite prevalence. Each of the malaria outcomes were examined for an association with *TAPBP* mRNA i-expression as a dichotomous variable with either *rs111686073* or *rs59097151* or a continuous variable with three levels of expression based on the mRNA data shown in Figure 1D (denoting *rs111686073/rs59097151* genotypes, respectively): CC/GG and CC/AG (lowest); CG/AG and CC/AA (intermediate); and CG/AA and GG/AA (highest).

| Malaria Incidence | IRRa | Lower Bound | Upper Bound | p | Empirical p | False Disc. Rate | N |
|---|---|---|---|---|---|---|---|
| Continuous *TAPBP* mRNA i-expression[c] | 0.833 | 0.691 | 1.004 | 0.055 | 0.026 | 0.110 | 833 |
| rs111686073 genotype[d] | 0.913 | 0.766 | 1.090 | 0.314 | 0.220 | 0.393 | 833 |
| rs59097151 genotype[e] | 0.868 | 0.745 | 1.011 | 0.068 | 0.046 | 0.159 | 833 |

| Parasite Prevalence | ORb | Lower Bound | Upper Bound | p | Empirical p | False Disc. Rate | N |
|---|---|---|---|---|---|---|---|
| Continuous TAPBP mRNA i-expression[c] | 0.722 | 0.573 | 0.911 | 0.006 | 0.002 | 0.011 | 835 |
| rs111686073 genotype[d] | 0.834 | 0.672 | 1.035 | 0.099 | 0.058 | 0.128 | 835 |
| rs59097151 genotype[e] | 0.781 | 0.644 | 0.946 | 0.012 | 0.007 | 0.027 | 835 |

**[a]Incidence Rate Ratio, [b]Odds Ratio, [c]*TAPBP* mRNA i-expression as a continuous variable with three levels of expression imputed from genetic data and mRNA expression in Figure 1d, d CG/GG vs. CC (high vs. low), e AA vs. AG/GG (high vs. low)**

**Table S4.** Tapasin dependence shows no consistent effect upon malaria outcomes. Each of the malaria outcomes were examined for an association with tapasin dependence as a continuous variable based upon the values shown in Figure S4, or as a dichotomous variable as shown in Figure S5. Results in parentheses are adjusted for B*53:01 for HLA-B, C*06:02 for HLA-C or both for HLA-ABC.

**Continuous tapasin dependence**

| Malaria Incidence | IRR[a] | Lower Bound | Upper Bound | p | Empirical p | False Disc. Rate | N |
|---|---|---|---|---|---|---|---|
| HLA-ABC | 1.168 | 0.943 | 1.448 | 0.154 | 0.091 | 0.291 | 833 |
| HLA-A | 1.071 | 0.855 | 1.342 | 0.549 | 0.524 | 0.742 | 833 |
| HLA-B | 1.066 | 0.933 | 1.217 | 0.347 | 0.265 | 0.466 | 833 |
| HLA-C | 1.176 | 0.882 | 1.567 | 0.269 | 0.199 | 0.375 | 833 |

| Parasite Prevalence | OR[b] | Lower Bound | Upper Bound | p | Empirical p | False Disc. Rate | N |
|---|---|---|---|---|---|---|---|
| HLA-ABC | 1.240 | 0.951 | 1.618 | 0.113 | 0.072 | 0.148 | 835 |
| HLA-A | 1.167 | 0.879 | 1.549 | 0.285 | 0.268 | 0.379 | 835 |
| HLA-B | 1.045 | 0.885 | 1.233 | 0.604 | 0.565 | 0.655 | 835 |
| HLA-C | 1.547 | 1.083 | 2.209 | 0.017 | 0.009 | 0.034 | 835 |

**Dichotomous tapasin dependence**

| Malaria Incidence | IRR[a] | Lower Bound | Upper Bound | p | Empirical p | False Disc. Rate | N |
|---|---|---|---|---|---|---|---|
| HLA-ABC | 1.037 (1.015) | 0.890 (0.851) | 1.209 (1.212) | 0.639 (0.867) | 0.588 | 0.786 | 833 |
| HLA-A | 0.991 | 0.826 | 1.190 | 0.923 | 0.922 | 0.985 | 833 |
| HLA-B | 1.008 (1.061) | 0.865 (0.905) | 1.175 (1.245) | 0.917 (0.465) | 0.904 | 0.985 | 833 |
| HLA-C | 1.100 (1.043) | 0.946 (0.874) | 1.280 (1.244) | 0.215 (0.642) | 0.165 | 0.339 | 833 |

| Parasite Prevalence | OR[b] | Lower Bound | Upper Bound | p | Empirical p | False Disc. Rate | N |
|---|---|---|---|---|---|---|---|
| HLA-ABC | 1.021 (0.954) | 0.843 (0.766) | 1.237 (1.190) | 0.829 (0.678) | 0.811 | 0.844 | 835 |
| HLA-A | 1.136 | 0.903 | 1.431 | 0.276 | 0.263 | 0.379 | 835 |
| HLA-B | 1.038 (1.132) | 0.858 (0.931) | 1.256 (1.377) | 0.701 (0.214) | 0.673 | 0.730 | 835 |
| HLA-C | 1.234 (1.154) | 1.023 (0.929) | 1.487 (1.434) | 0.028 (0.195) | 0.020 | 0.053 | 835 |

[a] Incidence Rate Ratio, [b] Odds Ratio

| Tapasin Dependence Cutoffs | |
|---|---|
| HLA-ABC | 1.7 |
| HLA-A | 1.1 |
| HLA-B | 1.4 |
| HLA-C | 0.8 |

**Table S5.** High *TAPBP* mRNA i-expression is associated with protection against malaria among subjects with tapasin-dependent HLA-I genotypes. Malaria incidence and parasite prevalence were examined for an association with *TAPBP* mRNA i-expression in the context of tapasin dependence. Expression was examined as either a dichotomous variable examining *rs111686073* or *rs59097151* or as a continuous variable with three levels of expression based on the mRNA data shown in Figure 1D (denoting *rs111686073/rs59097151* genotypes, respectively): CC/GG and CC/AG (lowest); CG/AG and CC/AA (intermediate); and CG/AA and GG/AA (highest). Tapasin dependence of the HLA-A/B/C, HLA-A, HLA-B, and HLA-C genotypes was examined dichotomously based on the divisions shown in Figure S5 (cut-offs notated in Table S2). Results in parentheses are adjusted for B*53:01 for HLA-B, C*06:02 for HLA-C or both for HLA-ABC.

| **Malaria Incidence** | | | **IRR[a]** | **Lower Bound** | **Upper Bound** | **p** | **Empirical p** | **False Disc. Rate** | **N** |
|---|---|---|---|---|---|---|---|---|---|
| **HLA-ABC** | **Dependent** | Continuous | 0.541 (0.532) | 0.402 (0.388) | 0.728 (0.730) | 5.03E-05 (9.08E-05) | <0.0001 | 0.001 | 328 |
| | | rs111686073 | 0.839 (0.870) | 0.633 (0.644) | 1.113 (1.175) | 0.224 (0.363) | 0.162 | 0.339 | 328 |
| | | rs59097151 | 0.557 (0.542) | 0.435 (0.417) | 0.713 (0.703) | 3.50E-06 (4.21E-06) | <0.0001 | 0.001 | 328 |
| | **Independent** | Continuous | 0.940 (0.932) | 0.755 (0.740) | 1.192 (1.175) | 0.651 (0.554) | 0.601 | 0.786 | 505 |
| | | rs111686073 | 0.921 (0.937) | 0.738 (0.749) | 1.149 (1.173) | 0.466 (0.572) | 0.371 | 0.610 | 505 |
| | | rs59097151 | 0.999 (0.970) | 0.828 (0.802) | 1.205 (1.173) | 0.990 (0.751) | 0.989 | 0.989 | 505 |
| **HLA-A** | **Dependent** | Continuous | 0.686 | 0.421 | 1.117 | 0.130 | 0.118 | 0.301 | 143 |
| | | rs111686073 | 0.859 | 0.569 | 1.296 | 0.469 | 0.451 | 0.677 | 143 |
| | | rs59097151 | 0.733 | 0.481 | 1.118 | 0.149 | 0.151 | 0.339 | 143 |
| | **Independent** | Continuous | 0.821 | 0.670 | 1.005 | 0.056 | 0.029 | 0.122 | 690 |
| | | rs111686073 | 0.897 | 0.737 | 1.091 | 0.278 | 0.185 | 0.362 | 690 |
| | | rs59097151 | 0.860 | 0.729 | 1.014 | 0.072 | 0.049 | 0.177 | 690 |
| **HLA-B** | **Dependent** | Continuous | 0.530 (0.527) | 0.396 (0.388) | 0.709 (0.715) | 1.96E-05 (4.03E-05) | <0.0001 | 0.001 | 332 |
| | | rs111686073 | 0.717 (0.859) | 0.537 (0.569) | 0.958 (1.296) | 0.024 (0.469) | 0.009 | 0.044 | 332 |
| | | rs59097151 | 0.610 (0.600) | 0.481 (0.469) | 0.772 (0.768) | 4.01E-05 (4.73E-05) | <0.0001 | 0.001 | 332 |
| | **Independent** | Continuous | 0.993 (0.984) | 0.786 (0.775) | 1.255 (1.248) | 0.955 (0.892) | 0.947 | 0.985 | 501 |
| | | rs111686073 | 1.021 (1.055) | 0.821 (0.845) | 1.270 (1.317) | 0.851 (0.637) | 0.823 | 0.985 | 501 |
| | | rs59097151 | 0.992 (0.961) | 0.817 (0.788) | 1.205 (1.173) | 0.939 (0.697) | 0.935 | 0.985 | 501 |
| **HLA-C** | **Dependent** | Continuous | 0.714 (0.715) | 0.568 (0.567) | 0.899 (0.900) | 0.004 (0.004) | 0.002 | 0.012 | 543 |
| | | rs111686073 | 0.866 (0.896) | 0.698 (0.720) | 1.076 (1.116) | 0.195 (0.329) | 0.115 | 0.301 | 543 |
| | | rs59097151 | 0.749 (0.736) | 0.617 (0.606) | 0.909 (0.893) | 0.003 (0.002) | 0.002 | 0.012 | 543 |
| | **Independent** | Continuous | 0.914 (0.919) | 0.660 (0.653) | 1.267 (1.294) | 0.590 (0.629) | 0.554 | 0.763 | 290 |
| | | rs111686073 | 0.798 (0.803) | 0.580 (0.572) | 1.099 (1.128) | 0.167 (0.205) | 0.116 | 0.301 | 290 |
| | | rs59097151 | 1.021 (1.019) | 0.788 (0.775) | 1.324 (1.39) | 0.873 (0.892) | 0.865 | 0.985 | 290 |
| **Parasite Prevalence** | | | **OR[b]** | **Lower Bound** | **Upper Bound** | **p** | **Empirical p** | **False Disc. Rate** | **N** |
| **HLA-ABC** | **Dependent** | Continuous | 0.492 (0.470) | 0.346 (0.323) | 0.700 (0.683) | 8.15E-05 (7.82E-05) | <0.0001 | 0.001 | 328 |
| | | rs111686073 | 0.770 (0.734) | 0.551 (0.514) | 1.075 (1.047) | 0.125 (0.087) | 0.072 | 0.148 | 328 |
| | | rs59097151 | 0.520 (0.515) | 0.386 (0.376) | 0.702 (0.705) | 1.83E-05 (3.61E-05) | <0.0001 | 0.001 | 328 |

| | | | | | | | | | |
|---|---|---|---|---|---|---|---|---|---|
| | **Independent** | Continuous | 0.794 (0.788) | 0.593 (0.588) | 1.064 (1.056) | 0.123 (0.110) | 0.083 | 0.163 | 507 |
| | | rs111686073 | 0.836 (0.852) | 0.634 (0.645) | 1.102 (1.126) | 0.204 (0.260) | 0.134 | 0.235 | 507 |
| | | rs59097151 | 0.862 (0.847) | 0.676 (0.664) | 1.100 (1.080) | 0.233 (0.181) | 0.193 | 0.318 | 507 |
| **HLA-A** | **Dependent** | Continuous | 0.504 | 0.285 | 0.892 | 0.019 | 0.012 | 0.037 | 143 |
| | | rs111686073 | 0.667 | 0.413 | 1.077 | 0.098 | 0.070 | 0.148 | 143 |
| | | rs59097151 | 0.625 | 0.380 | 1.026 | 0.063 | 0.053 | 0.128 | 143 |
| | **Independent** | Continuous | 0.741 | 0.576 | 0.953 | 0.020 | 0.008 | 0.031 | 692 |
| | | rs111686073 | 0.847 | 0.666 | 1.078 | 0.177 | 0.113 | 0.213 | 692 |
| | | rs59097151 | 0.795 | 0.645 | 0.980 | 0.031 | 0.018 | 0.050 | 692 |
| **HLA-B** | **Dependent** | Continuous | 0.470 (0.446) | 0.333 (0.310) | 0.664 (0.642) | 1.80E-05 (1.37E-05) | <0.0001 | 0.001 | 332 |
| | | rs111686073 | 0.649 (0.603) | 0.464 (0.420) | 0.907 (0.865) | 0.011 (0.006) | 0.003 | 0.018 | 332 |
| | | rs59097151 | 0.564 (0.558) | 0.424 (0.414) | 0.749 (0.752) | 7.88E-05 (1.24E-04) | <0.0001 | 0.001 | 332 |
| | **Independent** | Continuous | 0.843 (0.838) | 0.622 (0.618) | 1.143 (1.136) | 0.272 (0.254) | 0.211 | 0.336 | 503 |
| | | rs111686073 | 0.923 (0.959) | 0.698 (0.725) | 1.221 (1.269) | 0.576 (0.771) | 0.512 | 0.621 | 503 |
| | | rs59097151 | 0.871 (0.845) | 0.674 (0.653) | 1.127 (1.094) | 0.294 (0.202) | 0.243 | 0.364 | 503 |
| **HLA-C** | **Dependent** | Continuous | 0.579 (0.563) | 0.434 (0.422) | 0.772 (0.750) | 1.96E-04 (8.80E-05) | 1.00E-04 | 0.001 | 544 |
| | | rs111686073 | 0.753 (0.745) | 0.575 (0.567) | 0.986 (0.977) | 0.039 (0.034) | 0.015 | 0.044 | 544 |
| | | rs59097151 | 0.639 (0.622) | 0.499 (0.486) | 0.817 (0.795) | 3.71E-04 (1.49E-04) | 2.00E-04 | 0.001 | 544 |
| | **Independent** | Continuous | 0.850 (0.855) | 0.576 (0.573) | 1.253 (1.276) | 0.411 (0.443) | 0.365 | 0.455 | 291 |
| | | rs111686073 | 0.853 (0.802) | 0.588 (0.542) | 1.238 (1.186) | 0.404 (0.269) | 0.348 | 0.444 | 291 |
| | | rs59097151 | 0.917 (0.952) | 0.672 (0.691) | 1.252 (1.314) | 0.587 (0.766) | 0.553 | 0.655 | 291 |

[a] Incidence Rate Ratio, [b] Odds Ratio

**Table S6.** High *TAPBP* mRNA i-expression in individuals with tapasin-dependent HLA-I genotypes is as protective as tapasin-independent genotypes. Malaria outcomes were examined using a three-level categorical variable combining *TAPBP* mRNA i-expression level and tapasin dependence level: 1) high *TAPBP* mRNA i-expression as measured by *rs59097151* (AA) and HLA-I tapasin-dependent genotypes, 2) low *TAPBP* mRNA i-expression as measured by *rs59097151* (AG/GG) and HLA-I tapasin-dependent genotypes, and 3) -tapasin-independent genotypes. Tapasin dependence was examined for the combination of HLA-A, -B, and -C allotypes, as well as each independently (cut-offs notated in Table S2). Results in parentheses are adjusted for B*53:01 for HLA-B, C*06:02 for HLA-C or both for HLA-ABC.

| **Malaria Incidence** | | **IRR[a]** | **Lower Bound** | **Upper Bound** | **p** | **Empirical p** | **False Disc. Rate** | **N** |
|---|---|---|---|---|---|---|---|---|
| **HLA-ABC** | High Expression & Dependence vs. Independence | 0.903 (0.885) | 0.763 (0.734) | 1.068 (1.069) | 0.233 (0.206) | 0.166 | 0.339 | 833 |
| | Low Expression & Dependence vs. Independence | 1.428 (1.451) | 1.145 (1.133) | 1.782 (1.858) | 0.002 (0.003) | 0.001 | 0.010 | 833 |
| **HLA-A** | High Expression & Dependence vs. Independence | 0.925 | 0.752 | 1.138 | 0.460 | 0.429 | 0.677 | 833 |
| | Low Expression & Dependence vs. Independence | 1.198 | 0.870 | 1.648 | 0.268 | 0.298 | 0.506 | 833 |
| **HLA-B** | High Expression & Dependence vs. Independence | 0.893 (0.935) | 0.755 (0.786) | 1.056 (1.113) | 0.185 (0.451) | 0.127 | 0.310 | 833 |
| | Low Expression & Dependence vs. Independence | 1.342 (1.438) | 1.076 (1.141) | 1.675 (1.811) | 0.009 (0.002) | 0.006 | 0.038 | 833 |
| **HLA-C** | High Expression & Dependence vs. Independence | 1.015 (0.958) | 0.863 (0.795) | 1.195 (1.154) | 0.854 (0.651) | 0.832 | 0.985 | 833 |
| | Low Expression & Dependence vs. Independence | 1.292 (1.244) | 1.063 (1.001) | 1.572 (1.547) | 0.010 (0.049) | 0.007 | 0.041 | 833 |
| **Parasite Prevalence** | | **OR[b]** | **Lower Bound** | **Upper Bound** | **p** | **Empirical p** | **False Disc. Rate** | **N** |
| **HLA-ABC** | High Expression & Dependence vs. Independence | 0.892 (0.837) | 0.724 (0.64) | 1.098 (1.056) | 0.281 (0.134) | 0.243 | 0.364 | 835 |
| | Low Expression & Dependence vs. Independence | 1.443 (1.392) | 1.082 (1.014) | 1.924 (1.911) | 0.013 (0.041) | 0.010 | 0.035 | 835 |
| **HLA-A** | High Expression & Dependence vs. Independence | 1.013 | 0.782 | 1.312 | 0.922 | 0.914 | 0.914 | 835 |
| | Low Expression & Dependence vs. Independence | 1.603 | 1.054 | 2.436 | 0.027 | 0.033 | 0.085 | 835 |
| **HLA-B** | High Expression & Dependence vs. Independence | 0.905 (0.984) | 0.736 (0.796) | 1.113 (1.215) | 0.346 (0.878) | 0.310 | 0.422 | 835 |
| | Low Expression & Dependence vs. Independence | 1.475 (1.634) | 1.110 (1.219) | 1.961 (2.192) | 0.007 (0.001) | 0.005 | 0.022 | 835 |
| **HLA-C** | High Expression & Dependence vs. Independence | 1.100 (1.021) | 0.900 (0.813) | 1.344 (1.282) | 0.352 (0.860) | 0.325 | 0.425 | 835 |
| | Low Expression & Dependence vs. Independence | 1.571 (1.505) | 1.230 (1.149) | 2.007 (1.971) | 2.99E-04 (0.003) | 1.00E-04 | 0.001 | 835 |

[a] Incidence Rate Ratio, [b] Odds Ratio

**SI References**

1. N. Ludwig *et al.*, Distribution of miRNA expression across human tissues. *Nucleic acids research* **44**, 3865-3877 (2016).
2. A. A. Bashirova *et al.*, HLA tapasin independence: broader peptide repertoire and HIV control. *Proceedings of the National Academy of Sciences of the United States of America* 10.1073/pnas.2013554117 (2020).
